# Rethinking peptide developability with sequence-only models: interpretable screening of microplastic-binding peptides with gated query pooling

**DOI:** 10.1039/d6sc01486k

**Published:** 2026-05-25

**Authors:** Guangyao Chen, Fengqi You

**Affiliations:** a College of Engineering, Cornell University Ithaca NY 14853 USA fengqi.you@cornell.edu; b AI for Science Institute (CUAISci), Cornell University Ithaca NY 14853 USA; c Cornell AI for Sustainability Initiative (CAISI), Cornell University Ithaca New York 14853 USA

## Abstract

Designing peptides for microplastic targeting is intrinsically multi-objective: sequence motifs that promote adsorption to hydrophobic polymers frequently elevate developability risks, including hemolysis, non-specific adsorption, and poor aqueous solubility. In this paper, we show that accurate developability screening can be achieved from sequence alone by focusing on the readout that converts token-level foundation model representations into peptide-level decisions. We introduce gated query pooling (GQP), a lightweight, backbone-agnostic evidence-selection head that learns a small set of query vectors to extract complementary signals from protein language model embeddings and gates them adaptively per peptide. With a consistent evaluation protocol and identical splits for all methods, GQP with sequence-only backbones reaches 91.09%, 86.30%, and 75.56% accuracy on hemolysis, non-fouling, and solubility, respectively, outperforming representative sequence-only and AlphaFold-augmented Multi-Peptide baselines. Beyond predictive accuracy, attention diagnostics and controlled counterfactual substitutions enable residue-level, testable design rules that connect model outputs to actionable sequence edits. Finally, integrating these developability constraints with PepBD-derived affinity scores for polyethylene, polypropylene, and polyethylene terephthalate supports scalable multi-objective prioritization of microplastic-binding candidates and reveals non-fouling as a dominant feasibility bottleneck, with coarse-grained molecular dynamics triage providing complementary physical evidence supporting the plausibility of the PepBD-prioritized selections.

## Abstract 2

Gated query pooling gives peptide language models a sharper eye for function. Learned queries and gates distill sequence embeddings into signals that predict hemolysis, non-fouling behavior, and solubility.

## Graphical abstract

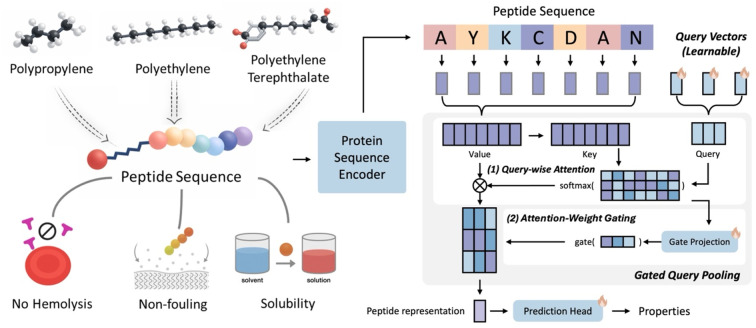

## Introduction

Microplastics have become a pervasive class of pollutants whose environmental fate is shaped by heterogeneous sources, transport pathways, and transformations across air, water, and soil. Recent syntheses emphasize that understanding microplastic pollution requires embracing this complexity, including polymer-specific behavior, weathering, and coupled physical–chemical processes that govern accumulation and exposure.^1–6^ Beyond ecological concerns, human exposure has also become an active area of investigation, with microplastics detected in biological samples such as the human placenta, underscoring the need for scalable mitigation and monitoring strategies.^7–9^

A promising direction is to develop molecular recognition elements that can selectively bind and capture microplastics.^10–13^ Peptides are attractive in this context because they are programmable, chemically diverse, and amenable to high-throughput synthesis and screening.^14^ Experimental studies have already demonstrated that engineered peptides can bind common plastics such as polypropylene (PP) and polystyrene (PS), enabling sensitive capture or biosensing of microplastics under relevant conditions.^15^ However, real-world pollution is inherently multiplastic, and dominant polymers such as polyethylene (PE), PP, and polyethylene terephthalate (PET) differ in surface chemistry and polarity.^11^ This motivates the adoption of design objectives that are plastic-specific and, ideally, applicable across multiple plastics. Recently, biophysical modeling frameworks such as PepBD have enabled large-scale computation of peptide adsorption to plastics, and protein language model-guided generative approaches have leveraged these scores to design high-affinity peptides for PE/PP/PET. Despite this progress, microplastic-binding peptide engineering is fundamentally multi-objective. Strong adsorption to hydrophobic polymers often favors hydrophobic and aromatic motifs, but the same features can increase nonspecific membrane interactions and compromise safety or formulation feasibility.^16^ In practice, candidate peptides must be screened not only for binding, but also for developability-related constraints such as low hemolysis, resistance to nonspecific adsorption (non-fouling), and sufficient aqueous solubility. In this work we treat these three properties as archetypal developability endpoints. The associated datasets, however, differ in sequence-length distributions and label construction, particularly for non-fouling, where negatives include insoluble and hemolytic peptides as well as scrambled positives. This creates a tension between function and biocompatibility that is difficult to resolve with single-objective optimization. The central challenge, therefore, is to couple plastic-specific affinity objectives with accurate, scalable developability prediction so that large libraries can be filtered down to candidates that are both high-affinity and biocompatible.^17^

Sequence-based machine learning has recently made this coupling feasible. Protein language models trained on large-scale sequence corpora can encode biophysical regularities directly from primary sequence. ProtTrans^18^ and ESM2 (ref. 19) are representative foundation encoders that have shown strong transfer to diverse protein prediction tasks and can recover structural and functional signals from sequence alone. Building on this foundation, PeptideBERT demonstrated that transformer-based, sequence-only models can predict key peptide properties, including hemolysis, non-fouling, and solubility, without explicit structural inputs.^18^ Multi-Peptide subsequently explored augmenting sequence models with predicted structural information through a language-graph framework, showing that structure-aware signals can improve selected settings.^20^ In parallel, AlphaFold has made accurate structure prediction broadly accessible, further catalyzing interest in structure-guided pipelines.^21^ Yet structure-augmented workflows, while potentially improving prediction in some settings, introduce additional computational stages and rely on imperfect structure predictions that can propagate uncertainty into downstream models. For high-throughput developability-oriented screening, this raises the practical question of when the added cost and complexity of structural modeling are justified relative to what can be achieved with sequence-only approaches.

In this paper, we rethink peptide developability prediction under a sequence-only paradigm and argue that a key bottleneck lies in the readout: how token-level representations are aggregated into a fixed-length peptide embedding for classification. In transfer learning settings with limited labeled peptides, this aggregation step can dominate performance, particularly when the task depends on localized sequence patterns rather than global composition alone. Because many peptide phenotypes are driven by sparse, localized motifs that reflect charge-hydrophobic patterning and amphipathic helical segments. Simple mean or max pooling can then dilute or mis-weight the decisive residues, especially for shorter peptides where single substitutions can have large effects.^22,23^ Motivated by cross-attention mechanisms that use learnable queries to extract evidence from variable-length inputs, we introduce gated query pooling (GQP) as a lightweight, backbone-agnostic readout for peptide property prediction. GQP learns a small set of query vectors that attend over token embeddings to extract complementary evidence. It then applies input-adaptive gating on the query-to-token attention weights (token-wise and query-wise) and pools the gated query summaries into a fixed-length representation. This evidence-selective design aims to maximize what can be extracted from sequence representations without requiring explicit 3D structure generation.

A second goal of this work is to connect model predictions to actionable design guidance. Attention maps provide useful diagnostic signals for how the readout routes evidence, and in our datasets, they recover chemically plausible residue-level tendencies, such as increased emphasis on hydrophobic and aromatic residues in hemolytic peptides, consistent with large-scale analyses of experimentally curated hemolysis data.^24^ For non-fouling, the diagnostics highlight mixed-charge and highly hydrated chemistries, aligning with experimental anti-biofouling measurements showing that zwitterionic peptide motifs built from EK and DK repeats strongly suppress protein adsorption and cell adhesion.^25^ For solubility, the same framework strongly solubilizes residues, consistent with mutational evidence that Asp, Glu, and Ser contribute particularly favorably to solubility compared with other hydrophilic residues.^26^ Because attention alone is not guaranteed to be a faithful explanation, we pair these diagnostics with controlled counterfactual substitutions that quantify how single-residue edits shift model outputs, yielding residue-level editing rules and a ranked notion of “intervenability” that is directly usable for sequence refinement. Finally, we integrate these developability predictors with PepBD-derived PE/PP/PET affinity scores to enable large-scale multi-objective prioritization of microplastic-binding peptides and to identify which constraints dominate feasibility at scale; notably, we find that non-fouling filtering removes the majority of high-affinity candidates, and plastic-specific substitution landscapes indicate that binding optimization is polymer-dependent, with larger edit sensitivities for PP and PET than for PE. These polymer-dependent patterns are mechanistically consistent with prior physics-based and AI-guided plastic-binding studies, which report that stronger PepBD scores are driven by increased van der Waals interactions and enrichment of bulky side chains (including aromatic residues such as tryptophan), and emphasize that sequence preferences differ across plastics. As a complementary physics-based sanity check, we additionally perform coarse-grained molecular dynamics (MD) triage on the PepBD-prioritized candidates. These simulations provide supporting physical evidence for the plausibility of our multi-objective selection under the tested proxy conditions.

In summary, our main contributions:

(1) Gated query pooling (GQP) improves sequence-only prediction of hemolysis, non-fouling, and solubility in both full-data and low-data settings.

(2) A systematic benchmark clarifies how backbone selection and adaptation strategy (frozen *versus* fine-tuned) shape transfer performance across developability tasks.

(3) Attention-based diagnostics summarize residue-level patterns and how evidence is routed through the GQP readout.

(4) Controlled counterfactual substitutions yield residue-level editing rules and intervenability rankings that translate predictions into actionable sequence edits.

(5) A developability-aware screening workflow integrates PepBD-derived PE/PP/PET affinity objectives with developability constraints to prioritize microplastic-binding candidates and highlights non-fouling as the dominant feasibility bottleneck.

(6) A unified coarse-grained molecular dynamics triage provides complementary physics-based evidence to de-risk the PepBD-prioritized candidate panel.

## Results

### Gated query pooling boosts sequence-only prediction of peptide developability

**Fig. 1:**
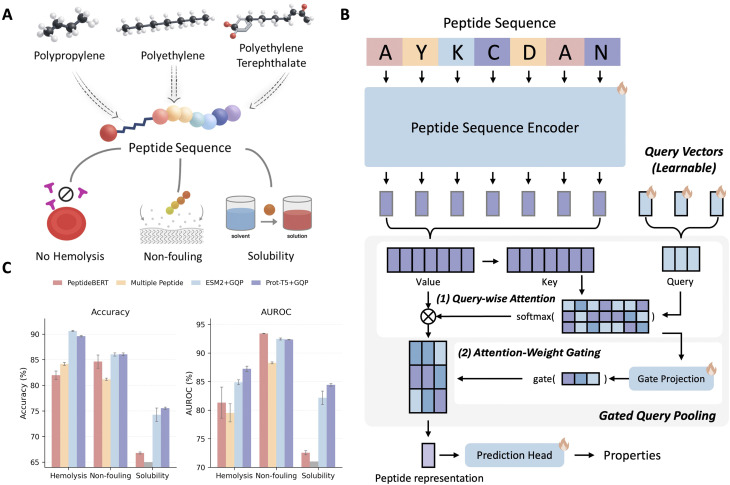
Sequence-only multi-property modelling enables developability-aware screening of microplastic-relevant peptides. (A) Conceptual workflow for identifying peptide candidates relevant to polypropylene (PP), polyethylene (PE) and polyethylene terephthalate (PET), while prioritizing three developability-related properties, no hemolysis, non-fouling and aqueous solubility. (B) Simplified overview of gated query pooling (GQP). A protein sequence encoder first produces residue-level token representations (keys and values). Learnable query vectors then summarize the sequence in two explicit steps: query-wise attention assigns each query to residue-level evidence, and attention-weight gating downweights weak or noisy query-token contributions before the gated summaries are pooled into a fixed-length peptide representation for property prediction. (C) Held out test accuracy (percent) for hemolysis, non-fouling and solubility across representative baselines (PeptideBERT,^18^ Multiple Peptide^20^) and sequence-only protein language model backbones equipped with GQP. Bars report mean accuracy (left) and mean AUROC (right). For the full-data ESM2 + GQP reruns, error bars indicate standard deviations across three independent random-seed runs (seeds 42, 43, and 44). N/A indicates results not reported for Multi Peptide on the solubility task.

Peptide developability is often constrained by safety and formulation requirements that can be assessed directly from sequence, including hemolysis, resistance to nonspecific adsorption (non-fouling), and aqueous solubility (Fig. 1A). Prior sequence-only work has shown that transformer protein language models can predict these properties from primary sequence, as exemplified by PeptideBERT,^18^ which fine-tunes ProtBERT^28^ for hemolysis, non-fouling, and solubility classification. Multi-Peptide^20^ extends this direction by combining a transformer sequence model with a graph neural network built from predicted structural information to model peptide properties. However, these results also highlight a practical bottleneck: performance is highly sensitive to how token-level representations are aggregated into a fixed-length peptide embedding, and structure-augmented pipelines^21,29^ introduce additional cost and potential error modes associated with structure prediction and cross-modal alignment.^20^ Here, we rethink that premise and show that, for developability screening, sequence-only foundation models can be highly accurate when paired with an evidence-selective readout.

To this end, we introduce gated query pooling (GQP), a lightweight, backbone-agnostic readout that converts token embeddings from a sequence encoder into a compact peptide representation using a small set of learnable query vectors. As illustrated in Fig. 1B, each query attends over the token sequence to form a query-specific summary, and an input-adaptive attention-weight gating mechanism modulates how each query routes evidence over tokens before pooling and prediction. This design is intended to separate sequence encoding from task-specific evidence extraction, allowing the model to learn multiple complementary “views” of a peptide and to down-weight uninformative queries for a given input. Importantly, GQP operates purely on sequence representations, making it compatible with widely used foundation protein language models such as ProtT5 (ref. 27) and ESM2.^19^ For fair comparison, we reimplemented both PeptideBERT^18^ and Multi-Peptide^20^ using the official code and trained them under the same benchmark split protocol as our models. Across the three developability tasks, adding GQP on top of sequence-only protein language model backbones achieves comparable or higher held-out accuracy relative to representative baselines (Fig. 1C). The same trend is observed for threshold-free discrimination, with ESM2 + GQP and ProtT5 + GQP also achieving strong AUROC across tasks (Fig. 1C). In particular, ESM2 + GQP reaches 90.37% accuracy for hemolysis and 86.00% for non-fouling, and ProtT5 + GQP reaches 75.54% for solubility, exceeding representative sequence-only and structure-augmented baselines where those results are available. Three independent full-data ESM2 + GQP reruns showed stable performance, supporting the robustness of the main benchmark comparison while avoiding a formal statistical-superiority claim for every endpoint. ProtT5 with GQP and ESM2 with GQP achieve consistently strong performance on hemolysis, non-fouling, and solubility, matching or exceeding prior sequence-only and structure-augmented baselines while avoiding the explicit generation of 3D structures. These results support a central premise of this work: for peptide developability screening, sequence-only foundation encoders can be highly effective when paired with an evidence-selective pooling head, and GQP provides a simple, general mechanism to realize that benefit in a plug-and-play manner across backbones.

### Backbone choice and adaptation strategy drive transfer-learning performance

**Fig. 2:**
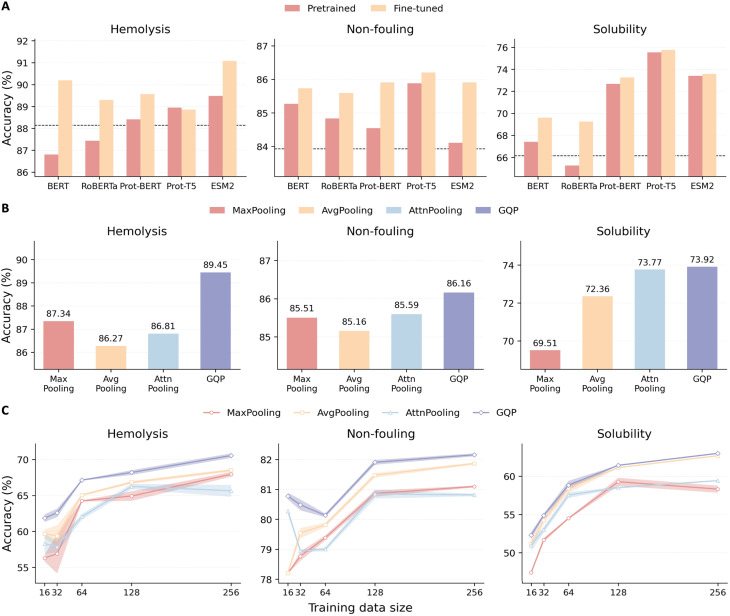
Backbone choice, adaptation strategy, and pooling head shape transfer performance and data efficiency. (A) Held out test accuracy for hemolysis, non-fouling, and solubility using different sequence encoders, comparing pretrained (frozen) features with fine-tuned encoders. The dashed line marks a reference baseline for each task. (B) Comparison of pooling heads on the same backbone and evaluation protocol, including max pooling, average pooling, attention pooling, and gated query pooling (GQP). (C) Accuracy as a function of training data size for each task, comparing pooling heads under matched subsampling of labeled peptides. Shaded regions indicate variability across repeated subsampling runs.

Sequence-only prediction of peptide developability depends not only on the downstream head but also on how well the pretrained encoder transfers to short, compositionally biased peptide sequences. To quantify this sensitivity, we benchmarked general-purpose language encoders (BERT^30^ and RoBERTa^31^) against protein-pretrained encoders spanning BERT- and T5-style architectures (Prot-BERT,^28^ Prot-T5,^27^ and ESM2 (ref. 19)) under two adaptation regimes: frozen feature extraction and end-to-end fine-tuning (Fig. 2A). Unless stated otherwise, fine-tuning refers to full end-to-end updates of all encoder parameters; we do not use partial unfreezing or LoRA.^32,33^ Protein-pretrained encoders consistently provide a stronger starting point, which is expected given that large-scale protein language modeling captures evolutionary and biophysical constraints directly from primary sequence.^28^ Across hemolysis, non-fouling, and solubility, fine-tuning improves performance over frozen encoders for most backbones, indicating that peptide property prediction benefits from adapting representations to the peptide domain shift and task-specific decision boundaries. The black dashed lines in Fig. 2A mark previously reported state-of-the-art accuracies on these benchmarks, including Multi-Peptide^20^ for hemolysis and PeptideBERT^18^ for non-fouling and solubility. Notably, under the same fine-tuning protocol, general NLP backbones (BERT and RoBERTa) achieve comparable or slightly higher accuracies on hemolysis and non-fouling than these reference baselines (Fig. 2A), whereas solubility remains best served by protein-pretrained backbones. This is plausible because peptide inputs are short sequences over a limited alphabet, and end-to-end fine-tuning plus an evidence-selective readout can adapt generic text encoders to peptide-specific local patterns.^18^

We also observe that solubility is more dependent on protein-specific pretraining than the other two tasks. In Fig. 2A, the strongest solubility results are achieved by protein-pretrained encoders (Prot-T5 and ESM2), whereas NLP backbones remain substantially behind even after fine-tuning. Moreover, fine-tuning yields only modest additional gains for solubility on the strongest protein backbones, suggesting diminishing returns once the encoder already captures relevant sequence-level biophysical features, while fine-tuning remains more beneficial for weaker or domain-mismatched backbones. These results motivate two practical conclusions for developability-oriented peptide screening. First, backbone selection matters and protein-pretrained encoders should be preferred when available. Second, adaptation strategy is a first-order design choice: freezing the encoder can be competitive in some settings, but fine-tuning generally yields more reliable improvements across tasks, mirroring the fine-tuning-centric approach used in peptide-specific transfer baselines such as PeptideBERT.^18^

### GQP outperforms standard pooling, especially in low-data regimes

A recurring bottleneck in peptide property transfer learning is the readout step that compresses token-level representations into a single peptide embedding. Simple permutation-invariant operators such as max and mean pooling are efficient but coarse, and can underutilize the structured information encoded by pretrained protein language models.^34^ Learnable attention-based pooling provides a more expressive alternative by allowing the model to select and aggregate evidence from different positions, rather than treating all tokens uniformly. In particular, GQP is closely related to seed or query-based attention pooling, where a small set of learnable query vectors aggregates variable-length inputs *via* attention.^34^ Consistent with this intuition, GQP achieves the strongest held-out accuracy across hemolysis, non-fouling, and solubility when compared with max pooling, average pooling, and attention pooling under the same backbone and evaluation protocol (Fig. 2B). Mechanistically, GQP forms multiple query-specific summaries *via* cross-attention and then applies input-adaptive gating on the attention weights (before renormalization) to modulate how evidence is routed before pooling. This gated aggregation increases the flexibility of the readout and enables sample-specific suppression of uninformative queries.^35^ Similar attention-weight gating designs can add useful nonlinearity and stabilize evidence selection in attention-based readouts and stability by introducing an additional nonlinearity on top of attention outputs, supporting the use of gating as a lightweight but effective enhancement to attention-based readouts. Importantly, GQP maintains a consistent advantage over standard pooling heads across training-set sizes, including in the low-data regime (Fig. 2C). To quantify performance under data scarcity, we construct reduced training sets by sampling *N* examples from the original training split, using label-stratified subsampling to preserve class balance. We repeat this procedure five times with independent random draws and keep all optimization and model hyperparameters fixed across *N*, so that changes in performance primarily reflect data availability rather than fraction-specific retuning. When training with limited labeled peptides, GQP consistently outperforms standard pooling heads at matched training set sizes, indicating a stronger inductive bias for extracting task-relevant evidence from pretrained token embeddings. To assess robustness under data scarcity, we repeated the low-data subsampling experiment five times with different random draws, and the observed gains of GQP remained stable across repeats. These results suggest that improving the readout is a high-leverage strategy for peptide developability prediction: with an evidence-selective, query-based pooling head, sequence-only backbones can translate pretrained representations into accurate predictions even when labeled data are limited.

### Attention patterns reveal residue-level drivers of peptide properties

**Fig. 3:**
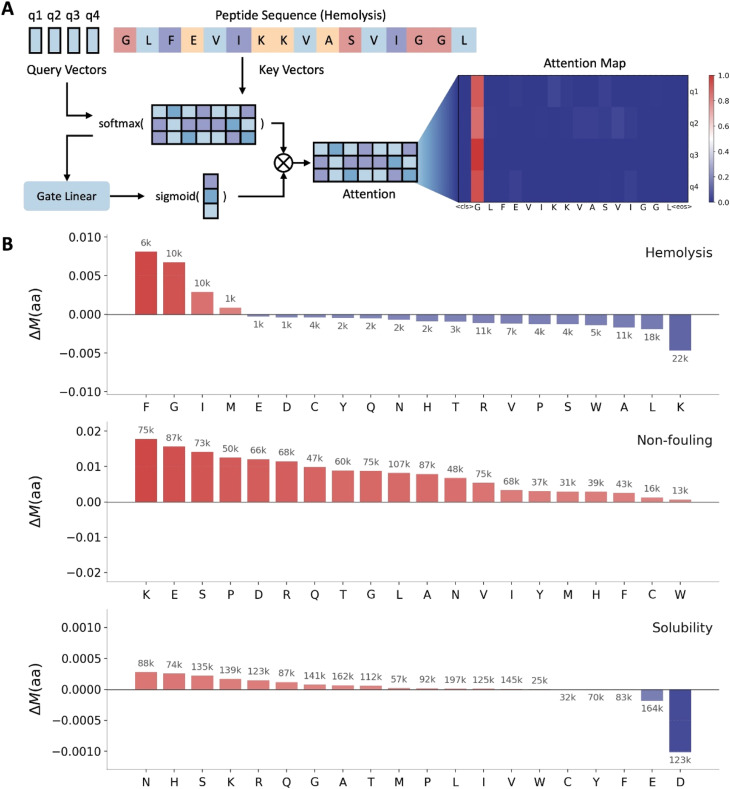
Attention-based interpretability of gated query pooling across peptide developability tasks. (A) Schematic of gated query pooling (GQP). Learnable query vectors q1 to q4 attend to token embeddings from the peptide sequence encoder to form query-specific summaries, which are modulated by multiplicative gating on attention weights (Gate Projection) before pooling. An example attention map is shown for a hemolysis peptide, with attention weights from each query over sequence tokens, including the special CLS and EOS tokens. (B) Amino acid level, frequency weighted attention mass differences for hemolysis, non-fouling, and solubility. For each task, Δ*M*(aa) is computed as *M*_positive_(aa) minus *M*_negative_(aa), where My(aa) denotes the mean gated attention mass assigned to residue aa in class y. Positive values indicate higher attention mass in the positive class, and negative values indicate higher attention mass in the negative class. Numbers above bars report the total residue counts *n* contributing to each amino acid aggregate.

**Fig. 4:**
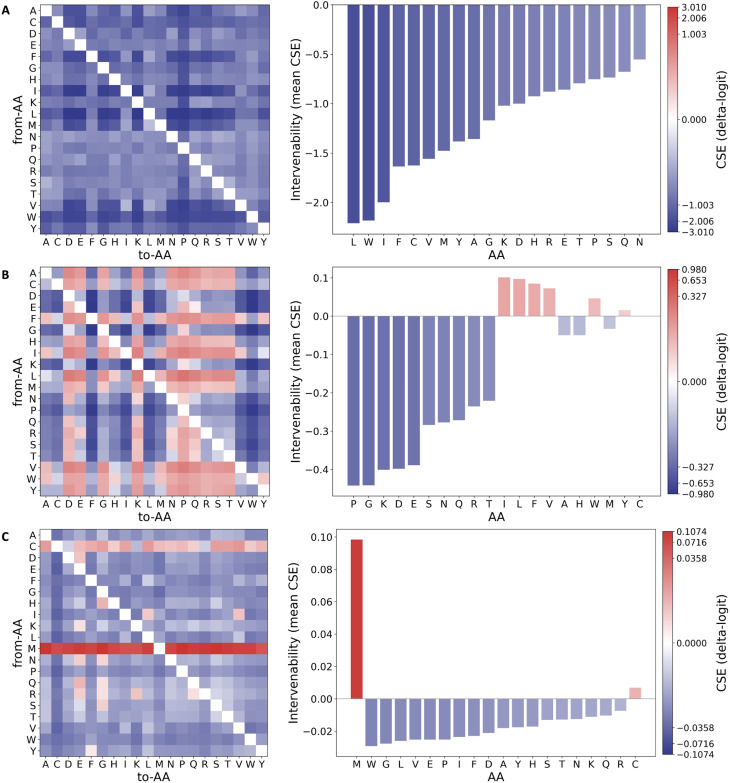
Controlled *in silico* residue substitutions quantify sequence-level drivers of peptide properties. (A–C) Left: controlled substitution effect (CSE; Δlogit) of single amino-acid substitutions (rows: from-AA, columns: to-AA), standardized across strata of measured global covariates (net charge, hydrophobic fraction, and length). Right: per-residue intervenability, defined as the mean CSE over substitutions from the same starting residue (ranked; colors follow the CSE scale). (A) Hemolysis. (B) Non-fouling. (C) Solubility. CSE is computed over all peptides in the dataset using a fixed trained model for post hoc interpretation.

Gated query pooling produces two complementary intermediate signals: query-wise attention over tokens (after gating and renormalization) and a derived token-level attention mass that summarizes where evidence is concentrated across queries (Fig. 3A). Because attention weights are not guaranteed to be faithful explanations of a model's decision process, we interpret these signals conservatively as diagnostic indicators of where the readout tends to route information. To summarize residue-level trends at scale, we compute a frequency-weighted attention mass *M*_*y*_(aa)for each class *y*, and report Δ*M*(aa) = *M*_1_(aa) − *M*_0_(aa) (Fig. 3B). All attention summaries exclude special tokens (*e.g.* [CLS], [SEP], padding) and are normalized over non-padding residues, ensuring comparability across backbones and sequence lengths. Positive values indicate residues that receive greater gated attention mass in the positive class, while negative values indicate residues emphasized in the negative class; counts above bars report the total number of residue instances contributing to each aggregate.^24^ Across tasks, the resulting Δ*M*(aa) profiles yield residue-level patterns that are consistent with known physicochemical drivers. For hemolysis, we observe that phenylalanine (F) is preferentially emphasized in the hemolytic class (that is, Δ*M*(F) > 0 in Fig. 3B under our label convention where the positive class denotes hemolytic peptides). This aligns with independent composition analyses reporting that hemolytic peptides are enriched in hydrophobic residues, including leucine and isoleucine, and to a lesser extent phenylalanine and other aromatic residues, whereas non-hemolytic peptides are enriched in positively charged residues such as lysine and arginine.^24^ In the same analysis, lysine is preferred at many positions in non-hemolytic peptides, highlighting that residue-level signals can differ systematically between hemolytic and non-hemolytic sequences. For non-fouling, charged and polar residues receive increased attention mass in the non-fouling class (Fig. 3B), consistent with experimental evidence that zwitterionic peptide sequences composed of paired oppositely charged residues such as EK, DK, ER, and DR repeats reduce protein adsorption and cell adhesion.^25^ For solubility, residues with acidic or strongly hydrophilic character are preferentially emphasized, consistent with measurements showing that aspartate (D), glutamate (E), and serine (S) contribute particularly favorably to solubility relative to other hydrophilic residues.^26^ Finally, we note that dataset construction can shape apparent residue-level “drivers.” PeptideBERT highlights that negative examples in the non-fouling dataset are largely associated with insoluble peptides, which can couple non-fouling and solubility signals and amplify charge- and hydration-related patterns.^18^ For this reason, we use the attention-derived trends in Fig. 3 primarily to generate hypotheses about residue-level cues and then validate them with controlled counterfactual analyses in later sections, which more directly test whether perturbing specific residues changes model outputs in the expected direction. Accordingly, we use Fig. 3 as a hypothesis-generating diagnostic rather than the main source of actionable interpretation, and place the intervention-based controlled substitution analysis in Fig. 4 as the primary evidence for residue-edit rules.

### Counterfactual residue substitutions yield actionable design rules

To translate model interpretation into actionable sequence edits, we performed controlled *in silico* mutagenesis and estimated the counterfactual effect of each single-amino-acid substitution, reported as the change in model log odds for the positive class (CSE, Δlogit; Fig. 4). Controlled effects are computed by stratifying sequences by net charge, hydrophobic fraction, and length using fixed bin widths (*q*_bin_ = 1.0, *h*_bin_ = 0.05, *l*_bin_ = 25) and standardizing across strata. Exact definitions are provided in Methods. The heat maps summarize substitution-specific effects for each from-AA → to-AA edit, while the bar plots quantify from-AA intervenability, computed as the mean CSE across all substitutions originating from the same residue. Because these effects are estimated with stratified control of global sequence properties (for example, net charge and hydrophobic fraction), the resulting patterns yield design rules that are less driven by background composition. This controlled substitution analysis is therefore the main basis for actionable residue-level design guidance because it directly measures model sensitivity to explicit single-residue perturbations rather than relying on attention weights alone.

For hemolysis (Fig. 4A), the most negative intervenability values are associated with strongly hydrophobic and aromatic residues, including L, I, W, and F, indicating that substitutions away from these residues tend to lower the hemolysis log odds on average. This agrees with large-scale analyses of experimentally validated hemolytic peptides, which report enrichment of leucine and isoleucine and, to a lesser extent, phenylalanine and tryptophan in hemolytic sequences relative to non-hemolytic controls.^36^ Accordingly, a practical rule to reduce hemolytic propensity is to target hydrophobic or aromatic hotspots (for example, L/I/F/W) for replacement with more polar or charged residues, consistent with the general link between hydrophobicity-driven membrane insertion and hemolysis.^36^ In addition, proline substitutions provide a mechanistically grounded option when the goal is to disrupt amphipathic helices, because proline is a potent α-helix breaker; experimental studies on model amphipathic peptides show that introducing or retaining a central proline can reduce membrane activity and hemolysis compared to helix-stabilizing variants.^37^ Non-fouling (Fig. 4B) effects separate residues whose substitution tends to decrease non-fouling propensity from those whose substitution tends to increase it. Residues with strongly negative mean CSE include K, D, and E, suggesting that these charged residues often support the non-fouling class and should be preserved when anti-adsorption is a priority. This is consistent with experimental anti-biofouling tests on zwitterionic peptide motifs, where surfaces presenting repeating units of EK and DK exhibit markedly reduced protein adsorption and cell adhesion compared with other charged pairings.^25^ Conversely, several hydrophobic residues show positive mean CSE (for example, I, L, F, V, and W), supporting a complementary rule of thumb for improving non-fouling behavior by reducing hydrophobic content or disrupting hydrophobic patches, which is consistent with hydration-based anti-fouling principles.^25^ Solubility (Fig. 4C) effects are smaller in magnitude than hemolysis and non-fouling, but show a clear dominant driver in our controlled analysis: methionine (M) exhibits a strongly positive intervenability, indicating that substitutions away from M tend to increase the solubility log odds. This direction is consistent with established solubility models that explicitly penalize hydrophobic residues and hydrophobic patches, including sequence-based solubility predictors such as CamSol^38,39^ and broader reviews of solubility-aware protein design.^38^ More broadly, experimental mutational analysis of RNase Sa shows that aspartate (D), glutamate (E), and serine (S) contribute particularly favorably to solubility, and are recommended targets for solubility-improving substitutions relative to other hydrophilic residues.^26^ Together with the strong M effect in Fig. 4C, these findings motivate a practical formulation rule: when solubility is limiting, prioritize substituting away from hydrophobic residues such as M and toward strongly solubilizing residues such as D, E, and S, subject to functional constraints.

Fig. 4 provides an editing playbook for multi-objective peptide design that is consistent with experimentally supported residue-level trends. Reduce hemolysis by mutating away from hydrophobic and aromatic residues (L/I/F/W) and, where appropriate, introducing helix-disrupting substitutions (for example, proline); improve non-fouling by preserving mixed-charge, highly hydrated motifs (notably K/E/D-rich patterns such as EK/DK); and increase solubility by prioritizing substitutions away from hydrophobic residues (highlighted by M) and toward solubilizing residues such as D, E, and S.

### Multi-objective screening identifies biocompatible microplastic-binding peptide candidates

**Fig. 5:**
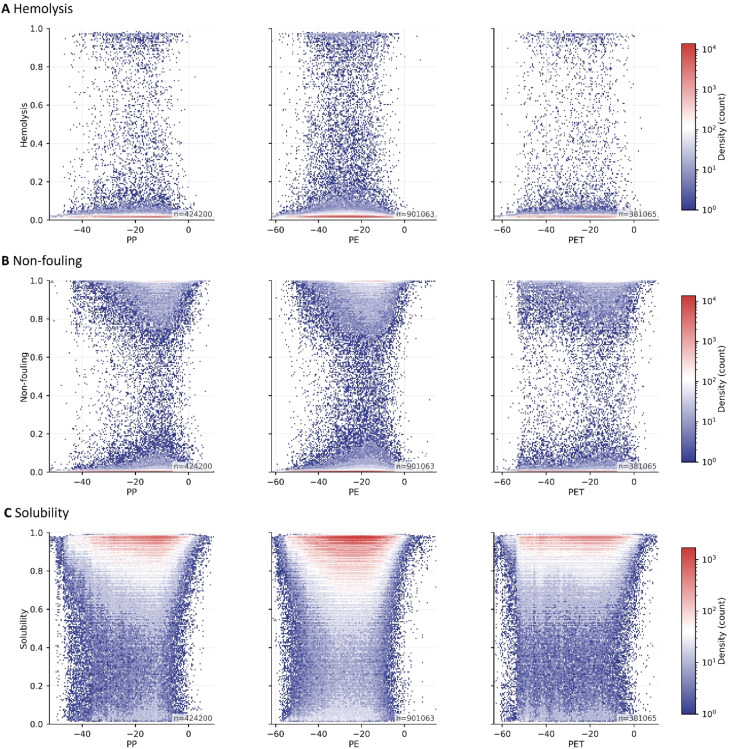
Relationship between microplastic-binding affinity and developability predictions across PP, PE, and PET peptide datasets. (A–C) Two-dimensional density plots showing the joint distribution between microplastic-binding affinity scores (*x* axis) and predicted developability probabilities (*y* axis) for hemolysis (A), non-fouling (B), and solubility (C). Each column corresponds to peptides evaluated for binding to polypropylene (PP), polyethylene (PE), or polyethylene terephthalate (PET). Colors indicate log-scaled point density (counts), and n denotes the number of peptide samples in each plastic-specific dataset. PP, PE, and PET affinity data are taken from the PepBD-based datasets.^40^

To connect microplastic targeting with peptide developability, we combined plastic affinity scores with the three developability classifiers (hemolysis, non-fouling, and solubility) and screened large peptide libraries for polyethylene (PE), polypropylene (PP), and polyethylene terephthalate (PET) derived from the PepBD resources.^40^Fig. 5 visualizes the resulting trade space by plotting predicted developability probabilities against PepBD affinity scores. Across all three plastics, the joint density plots show that high-affinity candidates (more favorable PepBD scores) are abundant, but high affinity alone does not guarantee biocompatibility: dense regions of the affinity distribution overlap with peptides predicted to be hemolytic, non-fouling negative, or poorly soluble.^15,41^ This observation is consistent with the broader microplastic binding peptide literature, which emphasizes that strong adsorption to hydrophobic polymer surfaces often arises from hydrophobic and aromatic sequence features that can also increase nonspecific membrane interactions and compromise safety if left unconstrained.^15,40^

**Fig. 6:**
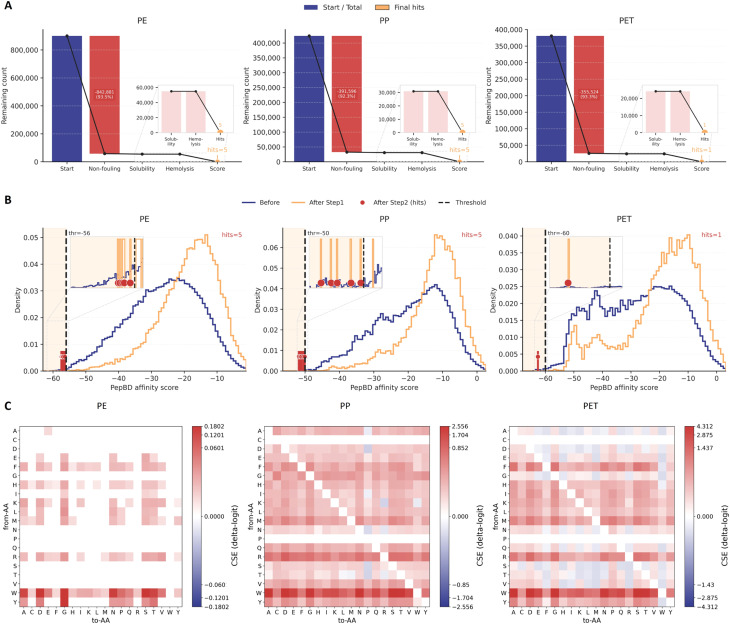
Multi-objective filtering identifies biocompatible microplastic-binding peptide candidates for PE, PP, and PET. (A) Sequential screening workflow applied to peptide libraries associated with polyethylene (PE), polypropylene (PP), and polyethylene terephthalate (PET). Bars report the number of peptides remaining after each developability filter (non-fouling, solubility, and non-hemolysis) and after ranking by the microplastic-binding score, with the final number of selected candidates (“final hits”) indicated for each plastic. (B) Density distributions of PepBD affinity scores for each plastic before screening (blue) and after applying the developability filters (orange). Dashed vertical lines mark the plastic-specific PepBD screening thresholds (lower is better): PE ≤ −56, PP ≤ −50, PET ≤ −60; red markers denote the final selected hits. (C) Controlled substitution effects (CSE, Δlogit) for the plastic-binding prediction objective, computed on the selected candidates, shown as from-amino-acid to to-amino-acid heat maps and summarized per plastic.

We therefore applied a sequential, multi-objective screening pipeline (Fig. 6A) that first enforces developability constraints and then enriches for the high-affinity tail of the PepBD score distribution. We use sequential filtering rather than Pareto ranking^42,43^ because the developability objectives are treated as hard feasibility constraints: peptides predicted to be hemolytic, insoluble, or fouling-prone are not actionable regardless of affinity. In large libraries, Pareto fronts can remain broad and may retain many high-affinity but infeasible sequences, whereas feasibility-first filtering yields a compact, interpretable feasible set before optimizing affinity within that set. PepBD scores are energy-like and span roughly −64 to +12 for 12-mers (lower is better).^44^ We therefore set plastic-specific cutoffs in the extreme negative tail, consistent with score ranges reported for top PepBD candidates in prior PepBD-based design studies. Specifically, Step 1 retains peptides that pass all three developability classifiers (non-fouling, solubility, and non-hemolysis) using fixed probability cutoffs. Step 2 then selects high-affinity candidates using plastic-specific PepBD score thresholds (lower is better): PE ≤ −56, PP ≤ −50, PET ≤ −60 (Fig. 6B). Starting from hundreds of thousands to nearly a million candidates per plastic, the non-fouling filter produces the largest initial reduction, followed by additional attrition from solubility and hemolysis constraints,^25^ yielding a compact feasible set before ranking by affinity (Fig. 6A). As shown by the largest drop in remaining candidates immediately after the non-fouling constraint across PE, PP, and PET, the majority of microplastic-binding candidates do not satisfy the anti-adsorption requirement before considering solubility or hemolysis. Importantly, the affinity distributions shift markedly after screening: compared to the “before” distribution, the “after” distribution concentrates near the extreme affinity region for each plastic, and the final selected hits lie beyond plastic specific score thresholds (Fig. 6B). This behavior indicates that the pipeline is not simply removing unsafe peptides, but is actively enriching for rare sequences that satisfy developability constraints while retaining strong predicted binding. Using these plastic-specific thresholds (PE ≤ −56, PP ≤ −50, PET ≤ −60), the final screens produce a small conservative candidate set, yielding five hits for PE, five hits for PP, and one hit for PET. We further assessed the sensitivity of these final hits to perturbations of the PepBD score thresholds in the SI.

Finally, we used residue substitution effect maps (CSE, Δlogit) to interpret and refine plastic binding within the screened set. Fig. 6C provides an interpretable, plastic-specific map of how single-residue edits are expected to shift the microplastic binding prediction within the screened candidate set. Across PP, PE, and PET, the substitution effect landscapes differ in both magnitude and pattern, indicating that the predicted binding objective is polymer-dependent rather than governed by a single, universal residue preference. Notably, the PP and PET maps show larger effect ranges and clearer residue class structure than the PE map, suggesting that, in the local neighborhood of our selected candidates, PP and PET binding predictions are more sensitive to single substitutions. Such sensitivity is mechanistically plausible because bulky hydrophobic and aromatic residues are repeatedly implicated as key contributors to adsorption on hydrophobic polymer surfaces, whereas hydrophilic residues tend to favor solvent exposure; for example, recent work on PepBD-guided microplastic binding design highlights bulky hydrophobic residues such as tryptophan and phenylalanine as strong contributors to plastic interactions and emphasizes polymer-dependent optimization.^16,45^ These CSE patterns offer an interpretable bridge between high-throughput screening and validation, analogous to recent microplastic-binding peptide studies that pair model-guided design with downstream experimental or mechanistic evaluation.

To further de-risk developability liabilities beyond sequence-level screening, we complemented the multi-objective selection with a physics-based sanity check using coarse-grained molecular dynamics (MD) simulations with the Martini 3 (ref. 46) force field and GPU-accelerated GROMACS.^47^ We evaluated the final candidate panel (11 screened candidates plus 2 controls) under a unified protocol across three proxy assays: membrane interaction as a hemolysis-risk proxy, multi-copy self-association in bulk water as a solubility proxy, and surface proximity as a non-fouling proxy. Across three independent replicates per peptide under fixed thermodynamic conditions and analysis thresholds, these MD proxies did not indicate strong membrane-active behavior, stable multi-copy aggregation, or adsorption events within the sampled trajectories, and provided consistent relative ordering among candidates. We therefore interpret the MD outcomes as relative physical triage evidence that complements the model-based screening, rather than definitive measurements of hemolysis, solubility, or anti-fouling performance. Full simulation setups, metrics, and summary statistics are reported in the Appendix (Fig. S10). The MD simulations should also be viewed as short-timescale triage rather than exhaustive sampling: the 0.5–1.0 µs production windows may miss slower peptide aggregation, membrane insertion, or surface-adsorption events that require longer simulations, enhanced sampling, or experimental validation.

## Discussion

Peptide design for environmental applications faces a practical bottleneck: candidate sequences must satisfy multi-property developability constraints, such as low hemolysis, non-fouling behavior, and sufficient solubility, while also meeting a target function such as polymer binding. Prior work has shown that sequence-only protein language models can predict several of these properties, and multimodal pipelines have explored incorporating predicted structure to improve selected tasks. Our results support a complementary, and in some settings simpler, conclusion. For developability-oriented screening, the limiting factor is often not the absence of explicit 3D structure, but the effectiveness of the readout that converts token-level representations into a peptide-level decision. This reframes the question posed in the title, namely, what must be added beyond sequence to predict peptide behavior, toward what must be learned to extract task-relevant evidence from sequence representations. Compared with PeptideBERT and Multi-Peptide, the closest sequence-only and structure-augmented references for these peptide-property benchmarks, GQP gives similar or better performance while remaining sequence-only. In Fig. 1c, ESM2+GQP improves accuracy over the best reported baseline by 2.22 percentage points for hemolysis and 2.07 percentage points for non-fouling; for solubility, where Multi-Peptide does not report a result, ESM2 + GQP and ProtT5 + GQP improve over PeptideBERT by 6.50 and 9.39 percentage points, respectively. These comparisons suggest that the main gain comes from how residue-level language-model features are read out, rather than from requiring predicted structures for every candidate. This is useful for large-scale screening because GQP is backbone-agnostic, avoids a structure-prediction step, and still provides attention and controlled-substitution diagnostics that can guide residue-level design hypotheses.

A key implication is that pooling is a high-leverage design choice for peptide transfer learning. Gated query pooling (GQP) implements a query-based evidence extraction interface that is conceptually related to learnable seed or query pooling mechanisms in attention-based set models, such as Pooling by Multihead Attention. In practice, this readout consistently improves accuracy and is most beneficial when labeled data are scarce, suggesting that a structured, evidence-selective head can compensate for limited supervision by learning where to attend within pretrained token representations rather than relying on coarse global statistics. This design also supports interpretability in a way that separates diagnostic signals from actionable, testable edits. Attention-derived summaries are useful for generating residue-level hypotheses, while controlled counterfactual substitution analyses directly quantify how model outputs change under single-residue edits while controlling for global composition. These developability models enable a multi-objective screening loop when paired with microplastic binding objectives. Using PepBD-derived PE, PP, and PET affinity scores, we find that high predicted affinity is abundant but frequently co-occurs with unfavorable developability predictions, motivating explicit constraint-based filtering rather than affinity-only selection. The sequential screen further indicates that non-fouling is the dominant feasibility bottleneck, consistent with the stringent hydration requirements needed to suppress nonspecific adsorption.

Several limitations define clear next steps, and they reflect different kinds of validity. First, the validity of benchmark developability prediction depends on how well benchmark labels and distributions match downstream use. We frame developability as binary classification, but real decisions often need calibrated probabilities or continuous readouts such as hemolysis intensity. Dataset construction can also couple properties. This can amplify correlated signals and reduce generalization. Future work should improve calibration and reduce confounding in dataset design. Second, the validity of our interpretability analyses depends on faithfulness and robustness. Attention patterns and controlled substitution effects offer plausible residue-level hypotheses. However, attention may not track causal evidence. Substitution effects can also change with the chosen controls or stratification. Sensitivity analyses and complementary faithfulness tests would strengthen these conclusions. Third, the validity of PepBD-derived binding hypotheses remains experimental. Our microplastic-binding candidates are computational hypotheses rather than validated leads. We provide mutation proposals to guide validation. However, adsorption strength and selectivity still need to be tested, especially on aged or biofilm-coated plastics. Biocompatibility also requires standardized assays and mechanistic follow-up. Despite these limitations, the broader message is that sequence-only foundation models can enable practical multi-objective peptide screening when the readout extracts task-relevant evidence.

More specifically, the microplastic screening component should be interpreted within the PE/PP/PET scope of the PepBD-derived datasets used here. We did not experimentally test binding on plastic substrates *in vitro*, and the prioritized peptides should therefore be viewed as computationally ranked candidates rather than experimentally validated plastic-binding leads. A direct follow-up validation campaign should synthesize the top candidates and controls, quantify adsorption to PE, PP, and PET films or particles using fluorescence-labelled peptide retention, QCM-D, or compatible surface-retention assays, and test wash-off stability and selectivity against non-plastic surfaces or serum/protein backgrounds. In parallel, solubility, hemolysis, and cytotoxicity assays should be used to verify developability before iterative model refinement. With respect to generalization, the available benchmarks support length-stratified evaluation but do not provide harmonized species-origin annotations or modification-type metadata. We therefore interpret the present GQP developability models as primarily applicable to linear peptide sequences composed of canonical amino acids and lying within the length and composition regimes represented in the benchmark training distributions. Predictions for species-shifted peptide families, d-amino-acid peptides, non-canonical residues, cyclized peptides, terminally modified peptides, or other chemically modified sequences should be treated as outside the validated scope unless supported by additional metadata-rich training data and task-specific validation.

## Method

### Data sources

#### Developability property datasets (hemolysis, non-fouling, solubility)

We used the three peptide property benchmarks popularized by PeptideBERT. Hemolysis labels were derived from DBAASPv3 (ref. 18) using an HC50 threshold below 100 µg mL^−1^. Labels are defined at the measurement level and then aggregated to the unique-sequence level for modeling. Duplicate sequences with conflicting labels across experiments were removed before constructing the train/test split, following the benchmark preprocessing described in the SI Methods. The resulting hemolysis dataset contains 9316 sequences with 19.6% positives and 80.4% negatives. Solubility labels were sourced from PROSO II and comprise 18 453 sequences with 47.6% positives and 52.4% negatives. The non-fouling dataset was constructed from prior antifouling work and contains 3600 positives and 13 585 negatives. Negatives include insoluble and hemolytic peptides as well as scrambled positives. The three tasks have markedly different sequence-length distributions, which we report in Fig. S1. Hemolysis is concentrated in short peptides with a median length of 17 aa. Non-fouling is strongly skewed toward short peptides with a median of 8 aa and a long tail to 198 aa. Solubility is dominated by longer sequences with a median of 143 aa and a maximum of 198 aa. We did not truncate sequences. All models used a maximum input length of 512 and no dataset sequence exceeded this limit. Sequences were padded as needed. For hemolysis and non-fouling, we used the sequence-disjoint 80/20 split protocol from Multi-Peptide to enable direct comparisons across methods. For solubility, we created a new sequence-disjoint 80/20 split because Multi-Peptide provides fixed splits only for hemolysis and non-fouling. Split construction details are provided in the SI Methods.

#### Microplastic binding datasets (PE, PP, PET)

Plastic affinity supervision was taken from PepBD-derived adsorption scores for polyethylene (PE), polypropylene (PP), and polyethylene terephthalate (PET) used in recent microplastic-binding peptide design studies. For large-scale screening, we used PepBD-derived datasets^40^ that contain on the order of hundreds of thousands of scored sequences per plastic; for example, one PepBD aggregation reports 715 509 sequences for PE, 433 488 for PP, and 441 978 for PET, with peptides represented as fixed-length 12-mers (excluding cysteine and proline in that resource).

#### Gated query pooling

Gated query pooling is a lightweight readout that turns token-level embeddings from a sequence encoder into a single fixed-length peptide representation using a small set of learnable “queries,” adding only 0.008 M parameters on top of the backbone. Let the encoder output token embeddings 
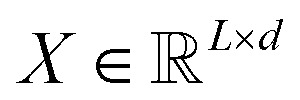
 for a peptide of length *L*, and let 
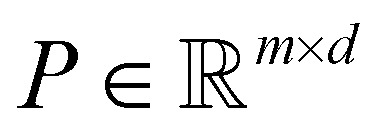
 denote *m* learnable query vectors. Each query is trained to extract a complementary “view” of the peptide by attending over all tokens. Unless otherwise noted, experiments use single-head query pooling with *m* = 4 learnable queries and full-softmax attention over all valid (non-padding) tokens. We fix the attention temperature to *τ* = 0.5, disable top-*k* sparsification, and do not use multi-head attention.

#### Query-to-token aggregation

Each query attends over all tokens and produces a query-specific summary as a weighted sum of token embeddings. We use the standard scaled dot-product attention form, which has been widely adopted in Transformer architectures.1
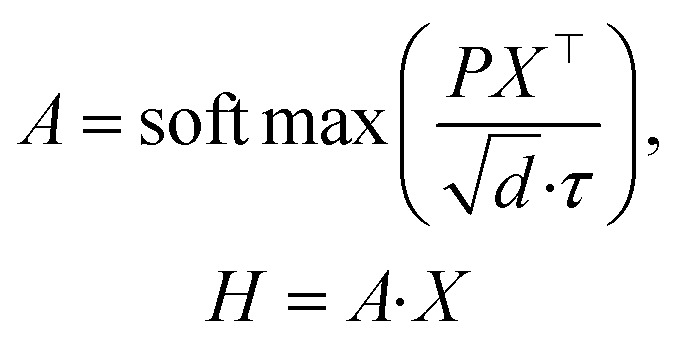
where 
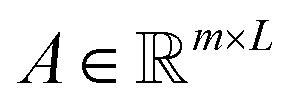
 is the attention weight matrix and 
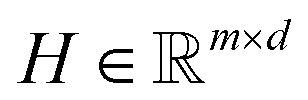
 contains one d-dimensional summary vector per query. In our implementation, the effective sharpness of attention is controlled by the temperature *τ*. In practice, padding positions (if any) are masked before the softmax so that attention is computed only over valid tokens.

#### Attention-weight gating

To enable input-adaptive suppression of uninformative evidence, GQP gates the attention weights directly (rather than gating the pooled embeddings). Specifically, we compute a token-wise gate 
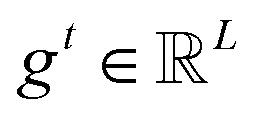
 from token embeddings and a query-wise gate 
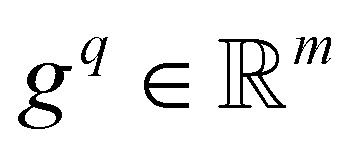
 from the learnable queries:

2 *g*^*t*^ = *ϕ*_*t*_(*X*), *g*_*q*_ = *ϕ*_*q*_(*P*)

In our implementation, both gates are identity-initialized multiplicative modulations parameterized by linear projections and a shared scalar gain initialized at zero, such that gating starts from 1 and is gradually learned. We then modulate attention weights multiplicatively and renormalize them so that each query's gated weights still sum to one across tokens:3
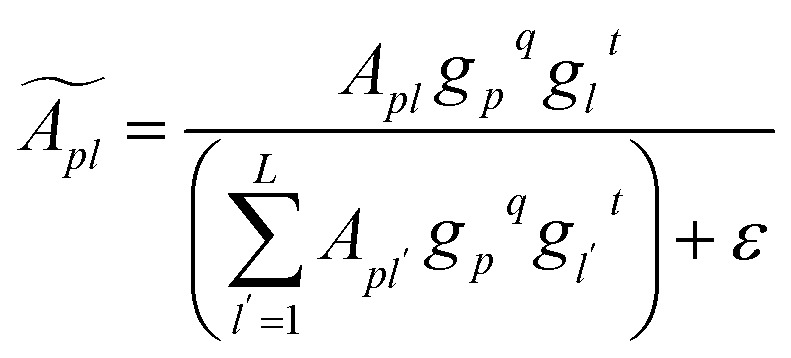
where *ε* is a small constant for numerical stability. We apply query-wise gating before renormalization for a unified multiplicative form; in practice, its effect is coupled with the nonlinearity/clamping and numerical stabilization. This formulation allows the model to down-weight specific tokens (*via g*^*t*^) and to modulate the contribution of entire query channels (*via g*^*q*^) while preserving the probabilistic structure of attention through renormalization. The gated query summaries are computed as:

4 *H̃* = *ÃX*.

#### Pooling to a peptide representation

Finally, we merge the gated query summaries into a single peptide embedding by averaging across queries:5
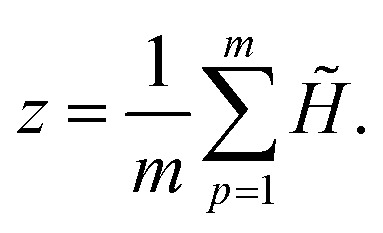


The resulting representation *z* is passed to a task-specific prediction head.

#### Attention-based diagnostics

To provide residue level diagnostics for gated query pooling (GQP), we record the query to token attention and the query gate for each input. We interpret these quantities conservatively as routing signals rather than definitive explanations, because multiple studies have shown that attention weights can be inconsistent proxies for feature importance and should not be treated as fail safe explanations.

#### Per query attention

For each peptide, GQP produces a post-gating attention matrix 
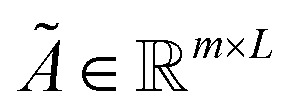
, where 
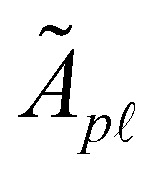
 is the gated-and-renormalized attention weight assigned by query *p*to token position 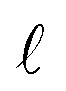. Padding positions (if any) are masked using the same convention as in GQP (*i.e.*, logits for padded positions are set to a large negative value before the softmax), so attention is normalized over valid (non-padding) tokens and 
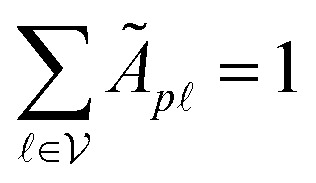
 for each query *p*. We visualize *Ã* as the per-query attention map in Fig. 3A.

#### Gated attention mass

To summarize where the readout allocates evidence after gating, we compute a token-level attention mass directly from the gated-and-renormalized attention weights. For a peptide, let 
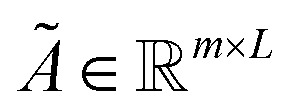
 denote the gated attention matrix produced by GQP (after masking and renormalization over valid tokens). We define the token-level mass as 
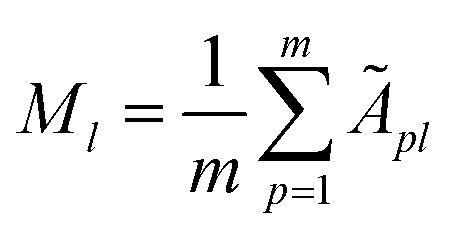
, which increases when multiple gated queries place probability mass on the same token position. If padding is present, we set *M*_*l*_ = 0for masked positions.

#### Amino acid level class contrast

For each task and class label *y* ∈ {0, 1}, we aggregate these masses over the dataset to obtain a frequency weighted amino acid summary. For amino-acid-level aggregation, we exclude padding and special tokens (CLS, SEP, and EOS) and only aggregate over residue positions that map to the 20 canonical amino acids. Special tokens can attract disproportionate attention mass and distort residue-level summaries. Let 
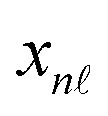
 be the amino acid identity at position 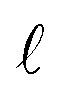 in peptide *n*. We compute6
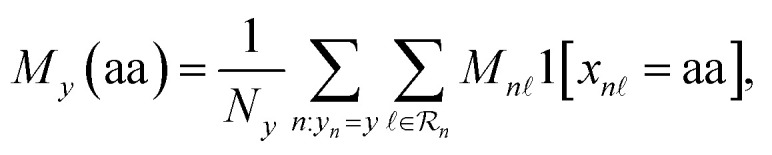
where *N*_*y*_ is the total number of residue positions (excluding padding and special tokens) in class *y*, and 
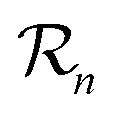
 denotes the set of such residue positions for peptide *n*. The reported class contrast is

7 Δ*M*(aa) = *M*_1_(aa) − M_0_(aa),

with positive values indicating residues receiving higher gated attention mass in the positive class. This construction explicitly accounts for residue frequency by normalizing by the total residue count per class.

#### Controlled counterfactual substitutions

To obtain actionable and testable edit rules, we perform single-residue *in silico* saturation mutagenesis and quantify substitution effects under a fixed trained predictor. This procedure is commonly used to interpret sequence-to-function models by perturbing inputs and measuring changes in model output.^48–50^

#### Controlled substitution effect (CSE, Δlogit)

For a peptide sequence *s* with model logit *f*(*s*) for the positive class, we define the substitution effect of changing position *i* from residue *a* to residue *a*′ as the logit difference:8
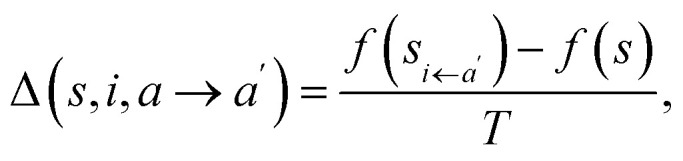
where 
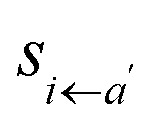
 denotes the mutated sequence. We report logit differences rather than probability differences because logits are additive and less sensitive to saturation near extreme probabilities. The temperature *T* is used only to scale logit differences; in practice we divide by max(*T*, 10^−3^) for numerical stability. Unless otherwise specified, we set *T* = 1.0 for all reported CSE results. At each position we evaluate all 19 non-identity substitutions (excluding *a* → *a*).

#### Sequence level aggregation to handle repeated residues

If a residue *a*appears multiple times within a peptide, treating each occurrence independently can over-weight sequences that contain many instances of *a*. We therefore treat the sequence as the unit of analysis. For each sequence and substitution *a* → *a*′, we average Δ(*s*, *i*, *a* → *a*′) over all positions *i* in the sequence where *x*_*i*_ = *a*. We then aggregate these sequence-level effects across sequences.

#### Standardized controlled effects by stratification

Substitution effects can reflect global sequence composition rather than residue-level drivers. To reduce this confounding, we compute controlled effects by stratifying sequences using global covariates and standardizing across strata. We stratify by net charge *Q*, hydrophobic fraction *H*, and sequence length *L*. Net charge is computed as *Q* = #(*K*, *R*) + 0.1 #(*H*) − #(*D*, *E*), with all other residues contributing 0. Hydrophobic fraction is defined as the fraction of residues in the hydrophobic set 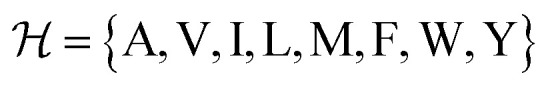. Strata are defined by discretizing these covariates using fixed bin widths. Specifically, we use a charge bin width of 1.0, a hydrophobic-fraction bin width of 0.05, and a length bin width of 25 amino acids. Each sequence is assigned to a stratum based on the resulting (*Q*, *H*, *L*) bin indices. Within each stratum *c*, we compute the mean sequence-level substitution effect Δ̂_*c*_(*a* → *a*′). We then form a standardized controlled effect by averaging stratum-specific estimates using empirical stratum weights. Importantly, weights are computed separately for each from-residue *a*, using only the subset of sequences that contain *a*. Denoting the corresponding stratum weights by *w*_*c*,*a*_, the controlled substitution effect is9
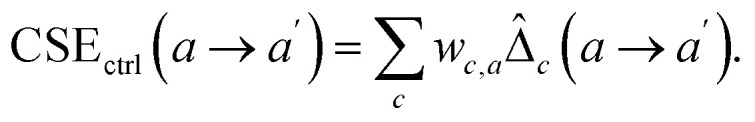


To ensure stable estimates, strata with fewer than five sequences are excluded and the remaining weights are renormalized to sum to one for each *a*. For each from-residue *a*, we exclude strata with fewer than five sequences containing *a*, and renormalize the remaining weights to sum to one. This procedure is a discrete form of standardization, or the g-formula, for estimating average effects under measured confounding.

#### Intervenability

To summarize which from-residues are most influential under substitution, we compute an intervenability score for each residue *a* as the mean controlled effect over all non-identity substitutions:10
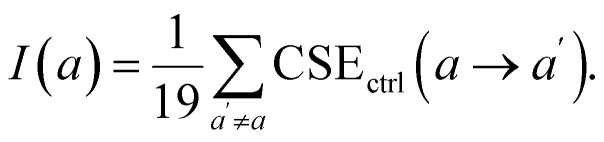


We exclude the identity substitution *a* → *a* and average over the remaining 19 substitutions. In Fig. 4, we visualize the full substitution matrices CSE_ctrl_(*a* → *a*′) (left) and the intervenability ranking (right) for hemolysis, non fouling, and solubility.

## Author contributions

F. Y. conceived the research; G. C. developed the models and analyzed the results; G. C. and F. Y. wrote the manuscript. All authors reviewed the final manuscript.

## Conflicts of interest

The authors declare no competing financial interests.

## Supplementary Material

SC-OLF-D6SC01486K-s001

## Data Availability

The computational models and data reported in this work are available under the MIT license at https://github.com/PEESEgroup/GQP. Supplementary information (SI): methodological details, diagnostic analyses, final peptide candidates, and MD-based triage results. See DOI: https://doi.org/10.1039/d6sc01486k.

## References

[cit1] Bank M. S., Mitrano D. M., Rillig M. C., Sze Ki Lin C., Ok Y. S. (2022). Nat. Rev. Earth Environ..

[cit2] Wang T., Zhao S., Zhu L., McWilliams J. C., Galgani L., Amin R. M., Nakajima R., Jiang W., Chen M. (2022). Nat. Rev. Earth Environ..

[cit3] Chen Q., Shi G., Revell L. E., Zhang J., Zuo C., Wang D., Le Ru E. C., Wu G., Mitrano D. M. (2023). Nat. Commun..

[cit4] Brahana P., Zhang M., Nakouzi E., Bharti B. (2024). Nat. Commun..

[cit5] Zhao S., Kvale K. F., Zhu L., Zettler E. R., Egger M., Mincer T. J., Amaral-Zettler L. A., Lebreton L., Niemann H., Nakajima R., Thiel M., Bos R. P., Galgani L., Stubbins A. (2025). Nature.

[cit6] Zhang Y., Wu P., Xu R., Wang X., Lei L., Schartup A. T., Peng Y., Pang Q., Wang X., Mai L., Wang R., Liu H., Wang X., Luijendijk A., Chassignet E., Xu X., Shen H., Zheng S., Zeng E. Y. (2023). Nat. Commun..

[cit7] Zhu L., Zhu J., Zuo R., Xu Q., Qian Y., An L. (2023). Sci. Total Environ..

[cit8] Ragusa A., Svelato A., Santacroce C., Catalano P., Notarstefano V., Carnevali O., Papa F., Rongioletti M. C. A., Baiocco F., Draghi S., D'Amore E., Rinaldo D., Matta M., Giorgini E. (2021). Environ. Int..

[cit9] Evangeliou N., Grythe H., Klimont Z., Heyes C., Eckhardt S., Lopez-Aparicio S., Stohl A. (2020). Nat. Commun..

[cit10] Guselnikova O., Trelin A., Kang Y., Postnikov P., Kobashi M., Suzuki A., Shrestha L. K., Henzie J., Yamauchi Y. (2024). Nat. Commun..

[cit11] Liu X., Wei W., Chen Z., Wu L., Duan H., Zheng M., Wang D., Ni B.-J. (2025). Nat. Water.

[cit12] Thompson R. C., Courtene-Jones W., Boucher J., Pahl S., Raubenheimer K., Koelmans A. A. (2024). Science.

[cit13] Microplastics are everywhere — we need to understand how they affect human health, Nat. Med., 2024, 30, 913 38641740 10.1038/s41591-024-02968-x

[cit14] Urso M., Ussia M., Novotný F., Pumera M. (2022). Nat. Commun..

[cit15] Woo H., Kang S. H., Kwon Y., Choi Y., Kim J., Ha D.-H., Tanaka M., Okochi M., Kim J. S., Kim H. K., Choi J. (2022). RSC Adv..

[cit16] Vendrell R. C., Ajagekar A., Bergman M. T., Hall C. K., You F. (2024). Sci. Adv..

[cit17] Dhoriyani J., Bergman M. T., Hall C. K., You F. (2025). PNAS Nexus.

[cit18] Guntuboina C., Das A., Mollaei P., Kim S., Barati Farimani A. (2023). J. Phys. Chem. Lett..

[cit19] Lin Z., Akin H., Rao R., Hie B., Zhu Z., Lu W., Smetanin N., Verkuil R., Kabeli O., Shmueli Y., Dos Santos Costa A., Fazel-Zarandi M., Sercu T., Candido S., Rives A. (2023). Science.

[cit20] Badrinarayanan S., Guntuboina C., Mollaei P., Barati Farimani A. (2025). J. Chem. Inf. Model..

[cit21] Jumper J., Evans R., Pritzel A., Green T., Figurnov M., Ronneberger O., Tunyasuvunakool K., Bates R., Žídek A., Potapenko A., Bridgland A., Meyer C., Kohl S. A. A., Ballard A. J., Cowie A., Romera-Paredes B., Nikolov S., Jain R., Adler J., Back T., Petersen S., Reiman D., Clancy E., Zielinski M., Steinegger M., Pacholska M., Berghammer T., Bodenstein S., Silver D., Vinyals O., Senior A. W., Kavukcuoglu K., Kohli P., Hassabis D. (2021). Nature.

[cit22] Alzain M., Daghistani H., Shamrani T., Almoghrabi Y., Daghistani Y., Alharbi O., Sait A., Mufrrih M., Alhazmi W., Alqarni M., Saleh B., Zubair M., Juma N., Niyazi H., Niyazi H., Halabi W., Altalhi R., Kazmi I., Altayb H., Ibrahem K., Alfadil A. (2025). Infect. Drug Resist..

[cit23] Hoang M., Singh M. (2025). Bioinformatics.

[cit24] Rathore A. S., Kumar N., Choudhury S., Mehta N. K., Raghava G. P. S. (2025). Commun. Biol..

[cit25] Chang R., Quimada Mondarte E. A., Palai D., Sekine T., Kashiwazaki A., Murakami D., Tanaka M., Hayashi T. (2021). Front. Chem..

[cit26] Trevino S. R., Scholtz J. M., Pace C. N. (2007). J. Mol. Biol..

[cit27] Heinzinger M., Weissenow K., Sanchez J. G., Henkel A., Mirdita M., Steinegger M., Rost B. (2024). NAR:Genomics Bioinf..

[cit28] Elnaggar A., Heinzinger M., Dallago C., Rehawi G., Wang Y., Jones L., Gibbs T., Feher T., Angerer C., Steinegger M., Bhowmik D., Rost B. (2022). IEEE Trans. Pattern Anal. Mach. Intell..

[cit29] Kovalevskiy O., Mateos-Garcia J., Tunyasuvunakool K. (2024). Proc. Natl. Acad. Sci. U. S. A..

[cit30] Devlin J. , Chang M.-W., Lee K. and Toutanova K., in Proceedings of the 2019 Conference of the North, Association for Computational Linguistics, Minneapolis, Minnesota, 2019, pp. 4171–4186

[cit31] Liu Y. , Ott M., Goyal N., Du J., Joshi M., Chen D., Levy O., Lewis M., Zettlemoyer L. and Stoyanov V., arXiv, 2019, preprint, arxiv.1907.11692, 10.48550/ARXIV.1907.11692

[cit32] Hu E. J. , Shen Y., Wallis P., Allen-Zhu Z., Li Y., Wang S., Wang L. and Chen W., arXiv, 2021, preprint, arxiv.2106.09685, 10.48550/ARXIV.2106.09685

[cit33] Mao Y., Ge Y., Fan Y., Xu W., Mi Y., Hu Z., Gao Y. (2025). Front. Comput. Sci..

[cit34] Lee J. , Lee Y., Kim J., Kosiorek A. R., Choi S. and Teh Y. W., in International conference on machine learning, 2019

[cit35] Qiu Z. , Wang Z., Zheng B., Huang Z., Wen K., Yang S., Men R., Yu L., Huang F., Huang S., Liu D., Zhou J. and Lin J., 2025

[cit36] Timmons P. B., Hewage C. M. (2020). Sci. Rep..

[cit37] Yang S., Lee J. Y., Kim H., Eu Y., Shin S. Y., Hahm K., Kim J. I. (2006). FEBS J..

[cit38] Sormanni P., Aprile F. A., Vendruscolo M. (2015). J. Mol. Biol..

[cit39] Sormanni P., Amery L., Ekizoglou S., Vendruscolo M., Popovic B. (2017). Sci. Rep..

[cit40] Wang S., Bergman M. T., Hall C. K., You F. (2025). J. Chem. Inf. Model..

[cit41] Motalebizadeh A., Fardindoost S., Hoorfar M. (2025). Trends Environ. Anal. Chem..

[cit42] Emmerich M. T. M., Deutz A. H. (2018). Nat. Comput..

[cit43] Deb K., Pratap A., Agarwal S., Meyarivan T. (2002). IEEE Trans. Evol. Comput..

[cit44] Jain V., Bergman M. T., Hall C. K., You F. (2025). Chem. Sci..

[cit45] Alshehri A. S., Bergman M. T., You F., Hall C. K. (2025). Digit. Discov..

[cit46] Risselada H. J. (2021). Nat. Methods.

[cit47] Abraham M. J., Murtola T., Schulz R., Páll S., Smith J. C., Hess B., Lindahl E. (2015). SoftwareX.

[cit48] Schreiber J., Nair S., Balsubramani A., Kundaje A. (2022). Bioinformatics.

[cit49] Nair S., Shrikumar A., Schreiber J., Kundaje A. (2022). Bioinformatics.

[cit50] Sasse A., Chikina M., Mostafavi S. (2024). iScience.

