# Supplementary material for: Rethinking peptide developability with sequence-only models: interpretable screening of microplastic-binding peptides with gated query pooling
Source: Chem Sci. 2026 May 25;17(25):12279–94. doi: 10.1039/d6sc01486k (PMC13223611; doi:10.1039/d6sc01486k)
Supplement: SC-017-D6SC01486K-s001 [file SC-017-D6SC01486K-s001.pdf]

Supplementary Information (SI) for Chemical Science.
This journal is © The Royal Society of Chemistry 2026

Supplementary Information for

Rethinking Peptide Developability with Sequence-Only Models: Interpretable Screening of Microplastic-Binding Peptides with Gated Query Pooling

Guangyao Chen[1,2], Fengqi You[1,2,3,†]

[1]College of Engineering, Cornell University, Ithaca, NY 14853, USA; [2]AI for Science Institute, Cornell University, Ithaca, NY 14853, USA; [3]Cornell AI for Sustainability Initiative (CAISI), Cornell University, Ithaca, NY 14853, USA; [†]Correspondence to: fengqi.you@cornell.edu

S1 Implementation Details 3

S2 Gated Query Pooling 7

S3 Attention-based Diagnostics 10

S4 Controlled Substitution Effects 13

S5 Attention Visualization Examples 14

S6 Residue-level Intervenability for PE, PP, and PET 15

S7 Final Selected Peptide Candidates 18

S8 GQP Code Availability and Usage 25

S1 Implementation Details

Datasets and preprocessing

Developability-related datasets. We considered three sequence-only developability predictors: hemolysis (`hemo`), non-fouling (`nf`), and solubility (`sol`). All three datasets were adopted from the PeptideBERT benchmark suite [1]. For `hemo` and `nf`, we followed the same train/test partition protocol as Multi-Peptide [2] to enable fair comparisons across methods. In particular, `hemo` sequences are derived from DBAASPv3 with experimentally annotated hemolysis labels. When multiple experimental measurements map to the same peptide sequence, we first aggregate labels at the sequence level. Sequences with conflicting binary labels across measurements are removed before splitting. The remaining dataset is partitioned into a non-overlapping training set (80%) and a held-out test set (20%) at the sequence level [2]. The `nf` dataset is constructed to predict resistance to non-specific interactions. Positives correspond to non-fouling peptides. Negatives include peptides associated with non-fouling failure modes, such as insoluble or hemolytic peptides. Negatives also include additional sequences generated following the protocol in Multi-Peptide [2]. The `nf` dataset is likewise split into 80% training and 20% test with no sequence overlap [2].

Cross-property coupling check for the non-fouling benchmark. Because the `nf` negative set includes insoluble and hemolytic peptides under the PeptideBERT/Multi-Peptide benchmark construction [1, 2], we performed an explicit post hoc coupling analysis to quantify whether `nf` labels are associated with independently trained hemolysis and solubility predictors. Using the final full-data GQP models trained with three random seeds (42, 43, and 44), we scored the combined `nf` training and validation sequences ($n = 22{,}927$; 9,348 `nf`-positive and 13,579 `nf`-negative sequences) with the hemolysis and solubility models. We then compared the distributions of predicted hemolysis probability, non-hemolysis probability, and solubility probability between `nf`-positive and `nf`-negative sequences. The three-seed mean predictions show that `nf`-positive sequences have lower hemolysis probability than `nf`-negative sequences (0.1003 vs. 0.3205), higher non-hemolysis probability (0.8997 vs. 0.6795), and higher solubility probability (0.8094 vs. 0.4929). The same pattern is supported by rank-based statistics: the best-oriented AUC is 0.8511 for hemolysis/non-hemolysis and 0.9026 for solubility, with Spearman correlations of -0.5977 for hemolysis probability, 0.5977 for non-hemolysis probability, and 0.6854 for solubility probability.

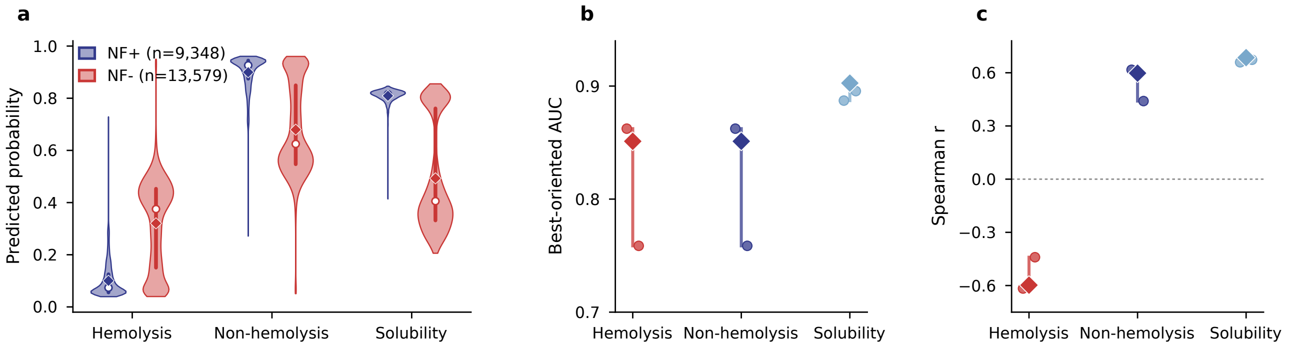

Figure S1: Quantitative evidence of cross-property coupling in the `nf` benchmark. **a**, Distributions of three-seed mean predicted probabilities for hemolysis, non-hemolysis, and solubility in `nf`-positive and `nf`-negative sequences. Diamonds indicate group means, open circles indicate medians, and vertical bars indicate interquartile ranges. **b**, Best-oriented AUC values quantifying how strongly each cross-property score separates `nf`-positive from `nf`-negative sequences across individual seeds. **c**, Spearman correlations between `nf` labels and cross-property scores across individual seeds. Circles denote individual seeds and diamonds denote the three-seed mean.

The analysis confirms measurable coupling in the benchmark labels: `nf`-positive sequences have lower predicted hemolysis probability and higher predicted solubility probability than `nf`-negative sequences. This coupling is consistent with the negative-sample construction inherited from the PeptideBERT/Multi-Peptide benchmark [1, 2], and it should be considered when interpreting high `nf` classification performance. Accordingly, we treat `nf` performance as benchmark performance under this established dataset definition rather than as evidence that the learned non-fouling signal is fully independent of hemolysis or solubility.

For `sol`, we adopted the PeptideBERT solubility dataset [1]. We created a sequence-disjoint 80/20 train/test split with label stratification and a fixed random seed. We did so because Multi-Peptide specifies a fixed split only for `hemo` and `nf`. Unless stated otherwise, all preprocessing and splitting were performed strictly at the sequence level to avoid leakage. All models used a maximum input length of 512 tokens. This exceeds the maximum peptide length in these benchmarks. Truncation was therefore never triggered. The sequence length distributions for each task and split are shown in Fig. S2. To ensure an apples-to-apples baseline, we re-implemented PeptideBERT on the same datasets using the official training recipe and hyperparameters reported by the authors [1]. Reported baseline numbers in this work correspond to our re-runs under the same data splits described above.

Full-data rerun uncertainty. To quantify run-to-run variability for the full-data benchmark, we retrained the final ESM2+GQP implementation using three independent random seeds (42, 43, and 44) under the same benchmark split protocol. The reruns showed stable best validation accuracy across hemolysis, non-fouling, and solubility. We therefore interpret the full-data comparison as supporting the main qualitative conclusion that GQP is comparable to or better than representative baselines in this benchmark setting, while avoiding a formal statistical-superiority claim for every individual endpoint.

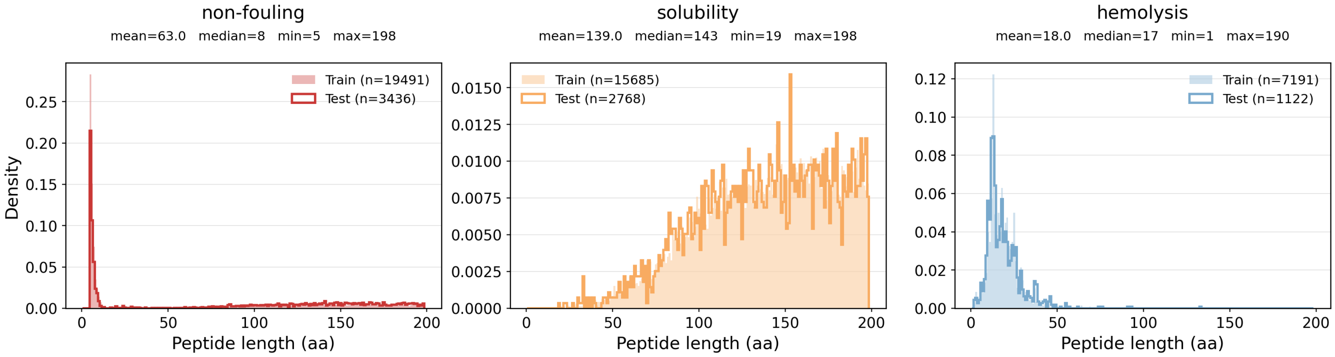

Figure S2: Sequence length distributions for the developability benchmarks. The histograms show peptide lengths in amino acids for the train and test splits of the non-fouling, solubility, and hemolysis datasets. Summary statistics are reported above each panel as mean, median, minimum, and maximum, and sample counts are shown in the legend. The train–test partitions are sequence-disjoint. No truncation was applied because all sequences are shorter than the 512-token model maximum length. Hemolysis and non-fouling are dominated by short peptides, while solubility is enriched for longer sequences.

Length-dependent performance and domain shift. To probe domain shift induced by differences in peptide-length distributions across datasets, we evaluate performance as a function of sequence length by binning the held-out test sets (Fig. S3). Several consistent patterns emerge. First, hemolysis shows relatively stable ranking performance across length bins: AUROC remains high with only modest variation, indicating that the classifier preserves discrimination quality across a wide length range. In contrast, threshold-dependent metrics vary more with length. F1 and AP are lowest in the shortest bin, improve in the mid-length bins, and then decline again in the longest bin, suggesting that short and very long hemolysis peptides are more challenging for fixed decision thresholds and precision–recall tradeoffs even when AUROC remains strong.

This behavior is consistent with the idea that hemolysis is driven by multiple interacting sequence features whose prevalence and strength can change with length. Second, non-fouling exhibits strong length sensitivity and clear small-sample effects. Performance is concentrated in the short-length regime where the benchmark provides most of its support, and both AUROC and AP peak in the second-shortest bin. However, the two longest non-fouling bins contain no positive examples, so AUROC is undefined and threshold-dependent metrics cannot demonstrate positive-class generalization in this regime. This indicates that the available non-fouling benchmark does not support claims of uniform transfer to longer positive non-fouling sequences, highlighting a data-coverage boundary when applying developability predictors outside their dominant length regime. Third, solubility is comparatively robust across long-length bins, but shows systematic gaps in F1 relative to AUROC and AP. Across the solubility bins, AUROC and AP remain consistently high with only mild variation, while F1 is noticeably lower and varies more with length. This pattern suggests that discrimination remains strong but that a single fixed threshold can yield length-dependent precision/recall tradeoffs, potentially reflecting changes in class prevalence or score calibration across length regimes. Across all tasks, we report N per bin to contextualize uncertainty, and bins with smaller support are expected to exhibit higher variance. Overall, these results support the claim that peptide-length distributions differ substantially across datasets and that length can modulate both discrimination and thresholded performance, motivating length-aware evaluation when transferring models across developability benchmarks.

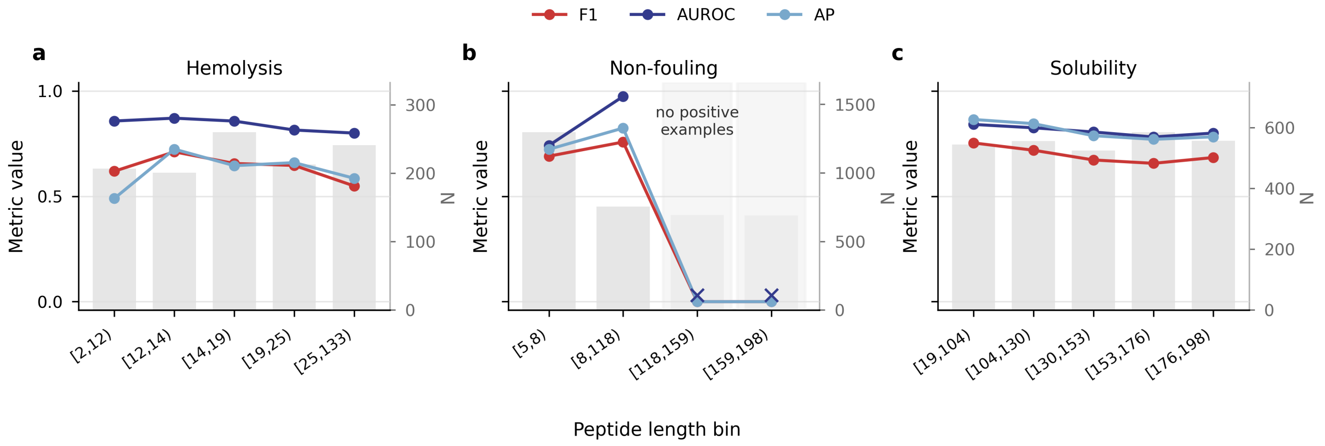

Figure S3: Performance versus peptide length bin on developability benchmarks. For each task (hemolysis, non-fouling, solubility), we partition the benchmark test set into length bins and report F1, AUROC, and average precision (AP) within each bin. Bars indicate the number of test sequences per bin (N, right axis). Length-dependent performance differences provide a simple diagnostic of domain shift arising from differing peptide length distributions across datasets. In the non-fouling panel, shaded long-length bins contain no positive examples; crosses indicate metrics that are undefined because only one class is present.

Applicable boundaries from generalization checks. The length-bin analysis provides a systematic validation axis for the available benchmarks, but it does not establish generalization across all peptide sources or chemical formats. The benchmark files used in this study do not provide harmonized species-origin annotations or modification-type metadata, so we cannot perform reliable species-stratified or modification-stratified validation from these datasets alone. Accordingly, the present GQP developability models should be interpreted as applying primarily to linear peptide sequences composed of canonical amino acids and lying within the length and composition regimes represented in the benchmark training distributions. Predictions for species-shifted peptide families, D-amino-acid peptides, non-canonical residues, cyclized peptides, terminally modified peptides, or other chemically modified sequences should

be treated as out-of-scope unless supported by additional metadata-rich training data and task-specific validation.

Microplastic-binding dataset. Microplastic-binding supervision was obtained entirely from the dataset reported in [3]. In that work, a pretrained protein language model is fine-tuned on biophysical modeling data of peptide adsorption to plastics generated by the PepBD algorithm. The fine-tuned model is then used to guide a generative model to design peptides with high affinity to multiple plastics, including polyethylene (PE), polypropylene (PP), and poly(ethylene terephthalate) (PET) [3]. Following their dataset definition, we performed secondary screening on the reported candidates and scores to construct our microplastic-binding evaluation set. We did not introduce any additional experimental labels. All binding-affinity supervision originates from the PepBD-based adsorption scoring pipeline used in [3].

OOD check for distribution shift in PepBD screening

To assess potential distribution shift when applying benchmark-trained developability classifiers to PepBD-generated fixed-length 12-mers, we performed an embedding-based out-of-distribution (OOD) check using k-nearest-neighbor (kNN) distance in representation space. For each developability task (hemolysis, non-fouling, solubility), we computed sequence embeddings using the same encoder and preprocessing used by the corresponding classifier, and built a kNN index on the benchmark training embeddings. For a query peptide, we define its OOD score as the distance to its k-th nearest neighbor in the benchmark training set. We then compare OOD score distributions between the benchmark held-out test set and a random sample of PepBD peptides pooled across PE/PP/PET (Fig. S4). As a conservative reference threshold, we report the 95th percentile of the benchmark test OOD scores, $q_{0.95}(\text{test})$, shown as a dashed vertical line in each panel.

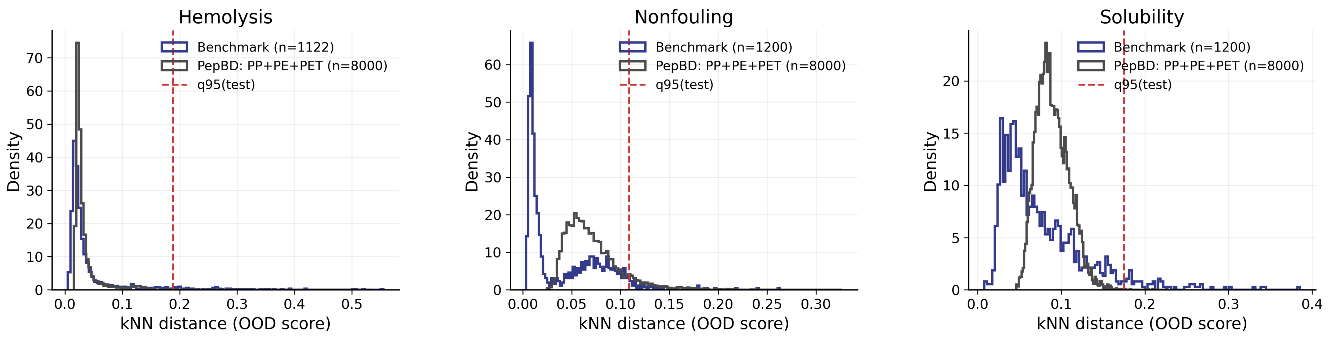

Figure S4: Embedding-based OOD check for PepBD-generated 12-mers. Distributions of kNN distance OOD scores for benchmark test peptides (blue) and a random sample of PepBD peptides pooled across PE/PP/PET (gray) for each developability task. The dashed red line denotes the 95th percentile of benchmark test OOD scores, $q_{0.95}(\text{test})$. Lower distances indicate greater similarity to the benchmark training distribution in embedding space.

Backbone encoders

Model choices. We evaluated five widely used pretrained encoders spanning both general-purpose text Transformers and protein language models (PLMs): BERT-large [4], RoBERTa-large [5], ESM2 (650M) [6], ProtBERT-BFD [7], and ProtT5-XL [7]. For transparency and reproducibility, the exact HuggingFace checkpoints, URLs, and the corresponding tokenization/input-formatting protocols are summarized in Supplementary Table S1.

Table S1: Tokenization protocols and links used in this work. "Spaced" indicates space-separated single-letter amino-acid tokens.

Backbone	Link	Input formatting	Tokenizer
`esm2_t33_650M_UR50D`	https://huggingface.co/facebook/esm2_t33_650M_UR50D	Raw amino-acid string (no inserted spaces).	ESM tokenizer via `from_pretrained()`.
`prot_bert_bfd`	https://huggingface.co/Rostlab/prot_bert_bfd	Uppercase; `U/Z/O/B` $\rightarrow$ `X`; **spaced** single-letter tokens.	ProtBERT tokenizer via `from_pretrained()`.
`prot_t5_xl_uniref50`	https://huggingface.co/Rostlab/prot_t5_xl_uniref50	Uppercase; `U/Z/O/B` $\rightarrow$ `X`; **spaced** single-letter tokens.	ProtT5 tokenizer via `from_pretrained()`.
`roberta-large`	https://huggingface.co/FacebookAI/roberta-large	**Spaced** single-letter tokens (after optional `U/Z/O/B` $\rightarrow$ `X`); whitespace-sensitive segmentation.	RoBERTa byte-level BPE tokenizer.
`bert-large-uncased`	https://huggingface.co/google-bert/bert-large-uncased	**Spaced** single-letter tokens; `U/Z/O/B` $\rightarrow$ `X`; then lowercase (uncased).	BERT tokenizer via `from_pretrained()`.

Tokenization and input formatting. For HuggingFace/Transformers backbones, we instantiate the model-specific tokenizer via `from_pretrained()` to match the pretrained vocabulary and special tokens. As summarized in Table S1, ESM2 consumes raw amino-acid strings, whereas ProtT5/ProtBERT and the text backbones operate on preprocessed, space-separated single-letter tokens (with `U/Z/O/B` $\rightarrow$ `X`; and additional lowercasing for uncased BERT to reduce `[UNK]`). Across all HuggingFace tokenizers, we enable padding, truncation, and special-token insertion; set the maximum length to `esm_max_len`; and request the special-token mask so that pooling ignores special tokens. Tokenization outputs are cached in memory per preprocessed sequence string.

S2 Gated Query Pooling

Gated Query Pooling (GQP) is an attention-based sequence pooling operator that produces a fixed-dimensional representation from variable-length token embeddings using a small set of learnable query vectors. The design follows the general idea of learnable "seed/query" vectors that summarize a set/sequence via cross-attention (e.g., Pooling by Multihead Attention in Set Transformer and query-based decoding in Perceiver-style models), but we introduce an optional gate on the attention weights to adaptively suppress uninformative tokens and queries.

In GQP, the protein sequence encoder first converts each residue into a token embedding. The learnable query vectors can be viewed as evidence detectors: each query searches the encoded peptide for a different type of sequence signal. Query-wise attention assigns weights from each query to all residue tokens, yielding query-specific summaries. The attention-weight gate then rescales these weights so that weak or noisy query-token contributions are reduced before pooling. The gated query summaries are averaged into a fixed-length peptide representation and passed to the classifier.

Inputs and parameters. Given token embeddings $\mathbf{X} \in \mathbb{R}^{B\times L\times D}$ and a binary token mask $\mathbf{M} \in \{0,1\}^{B\times L}$ (1 =valid amino-acid token, 0 =padding/special tokens), GQP uses P learnable queries $\mathbf{P} \in \mathbb{R}^{P\times D}$. We

```python
def gqp(X, M, P, T=0.5):
    # X: (B, L, D), M: (B, L), P: (Q, D)
    eps = 1e-12

    # 1) Prompt-query attention
    S = einsum("qd,bld->bql", P, X)
    S = where(M[:, None, :] > 0, S, -inf)
    S = S / max(T, 1e-3)
    A = softmax(S, dim=-1)  # (B, Q, L)

    # 2) Apply gate on attention weights
    tok_raw = token_gate_linear(X).transpose(1, 2)  # (B, 1, L)
    qry_raw = query_gate_linear(P).transpose(0, 1)  # (1, Q)

    # 3) Identity-initialized multiplicative gate
    #    gate_gain initialized to 0 => gate starts at 1
    gain = tanh(gate_gain)  # scalar
    tok_gate = clamp_min(1.0 + gain * tanh(tok_raw), 0.0)  # (B, 1, L)
    qry_gate = clamp_min(1.0 + gain * tanh(qry_raw), 0.0)  # (1, Q)

    A_g = A * tok_gate * qry_gate[:, :, None]  # (B, Q, L)
    A_g = A_g / clamp_min(A_g.sum(dim=-1, keepdim=True), eps)

    # 4) Weighted pooling and classifier
    H = einsum("bql,bld->bqd", A_g, X)  # (B, Q, D)
    z = H.mean(dim=1)  # (B, D)
    logits = mlp(z)

    return logits, A_g

```

Listing 1: Gated Query Pooling.

optionally apply (i) temperature scaling T to control attention sharpness, (ii) top-k sparsification to restrict each query to a small subset of tokens, and (iii) a lightweight multiplicative gate, parameterized by a token-wise projection, a query-wise projection, and a scalar gain, to modulate the attention weights before pooling. A concise implementation is shown below.

GQP attention and pooling. For each sequence b and query p, we first compute dot-product scores over token positions:

$$S_{bpl} = \langle \mathbf{P}_{p:}, \mathbf{X}_{bl:} \rangle \,, \qquad \mathbf{S} \in \mathbb{R}^{B \times P \times L}.$$

We apply masking by setting $S_{bpl} = -\infty$ wherever $\mathbf{M}_{bl} = 0$, ensuring padded and special tokens do not contribute to pooling:

$$S_{bpl} \leftarrow \begin{cases} S_{bpl}, & \mathbf{M}_{bl} = 1, \\ -\infty, & \mathbf{M}_{bl} = 0. \end{cases}$$

We then apply temperature scaling:

$$\tilde{S}_{bpl} = \frac{S_{bpl}}{\max(T, 10^{-3})}.$$

If top-k sparsification is enabled, we further restrict each query to its top-k tokens:

$$\bar{S}_{bpl} = \begin{cases} \tilde{S}_{bpl}, & l \in \mathrm{TopK}_k\big(\tilde{S}_{bp:}\big), \\ -\infty, & \text{otherwise.} \end{cases}$$

The (ungated) attention weights for each query are then computed with a softmax over token positions:

$$A_{bpl} = \mathrm{softmax}_l(\bar{S}_{bp:}), \qquad \mathbf{A} \in \mathbb{R}^{B\times P\times L}.$$

Gating on attention weights. We modulate the attention weights with token-wise and query-wise gates. Let $\mathbf{w}_{\text{tok}}, \mathbf{w}_{\text{qry}} \in \mathbb{R}^D$ and $b_{\text{tok}}, b_{\text{qry}} \in \mathbb{R}$ be the parameters of two shallow linear projections corresponding to `token_gate_linear` and `query_gate_linear` in Listing 1. For each token and query we compute

$$r_{bl}^{\text{tok}} = \mathbf{w}_{\text{tok}}^{\top}\mathbf{X}_{bl:} + b_{\text{tok}}, \qquad r_p^{\text{qry}} = \mathbf{w}_{\text{qry}}^{\top}\mathbf{P}_{p:} + b_{\text{qry}}.$$

Let θ be a learned scalar parameter (`gate_gain` in the code), and define

$$\gamma = \tanh(\theta) \in (-1, 1).$$

The token-wise and query-wise gates are then

$$g_{bl}^{\text{tok}} = \max\Big(1 + \gamma\ \tanh(r_{bl}^{\text{tok}}),\ 0\Big), \qquad g_p^{\text{qry}} = \max\Big(1 + \gamma\ \tanh(r_p^{\text{qry}}),\ 0\Big).$$

We apply these gates multiplicatively to the attention weights, renormalize, and obtain gated-pooled representations as

$$\hat{A}_{bpl} = A_{bpl}\, g_{bl}^{\text{tok}}\, g_p^{\text{qry}}, \qquad A_{bpl}^{\text{g}} = \frac{\hat{A}_{bpl}}{\sum_{l'=1}^{L}\hat{A}_{bpl'} + \varepsilon}, \quad \varepsilon > 0.$$

$$\mathbf{H}_{bp:} = \sum_{l=1}^{L} A_{bpl}^{\text{g}}\,\mathbf{X}_{bl:}, \qquad \mathbf{H} \in \mathbb{R}^{B\times P\times D}.$$

Intuitively, the gates g_{bl}^{tok} and g_p^{qry} enable the model to down-weight uninformative tokens and queries while starting close to standard query-based pooling when $\theta \approx 0$ (so that $g_{bl}^{\text{tok}} \approx g_p^{\text{qry}} \approx 1$).

Merging queries and prediction head. We merge the P query outputs into a single pooled embedding using mean aggregation:

$$\mathbf{z}_b = \frac{1}{P}\sum_{p=1}^{P}\mathbf{H}_{bp:} \in \mathbb{R}^D.$$

In our current implementation, mean merging is used regardless of the `pool_concat` switch to keep the dimensionality fixed across configurations; if additional sequence-level features $\mathbf{E} \in \mathbb{R}^{B\times D_{\text{extra}}}$ are provided, we concatenate them to form $\mathrm{concat}(\mathbf{z}_b, \mathbf{E}_b)$ before feeding the final vector into a task-specific MLP to obtain logits.

Ablation of Gated Query Pooling. To assess the effect of the gating mechanism and the number of query vectors in Gated Query Pooling, we performed an ablation study (Fig. S5). Panel A compares models with the gate disabled versus enabled, keeping all other settings fixed. The gated variant achieves slightly higher accuracy and AUROC and a noticeably larger PR-AUC, indicating that gating helps suppress uninformative tokens and improves calibration of the positive class. Panel B sweeps the number of query vectors $P \in \{1, 2, 4, 8, 16, 32, 64, 128\}$. All three metrics are relatively stable across this range, with PR-AUC peaking around $P = 4$. We therefore use $P = 4$ query vectors as the default configuration in all experiments. All variants in this ablation are trained end-to-end with the same optimization and data settings.

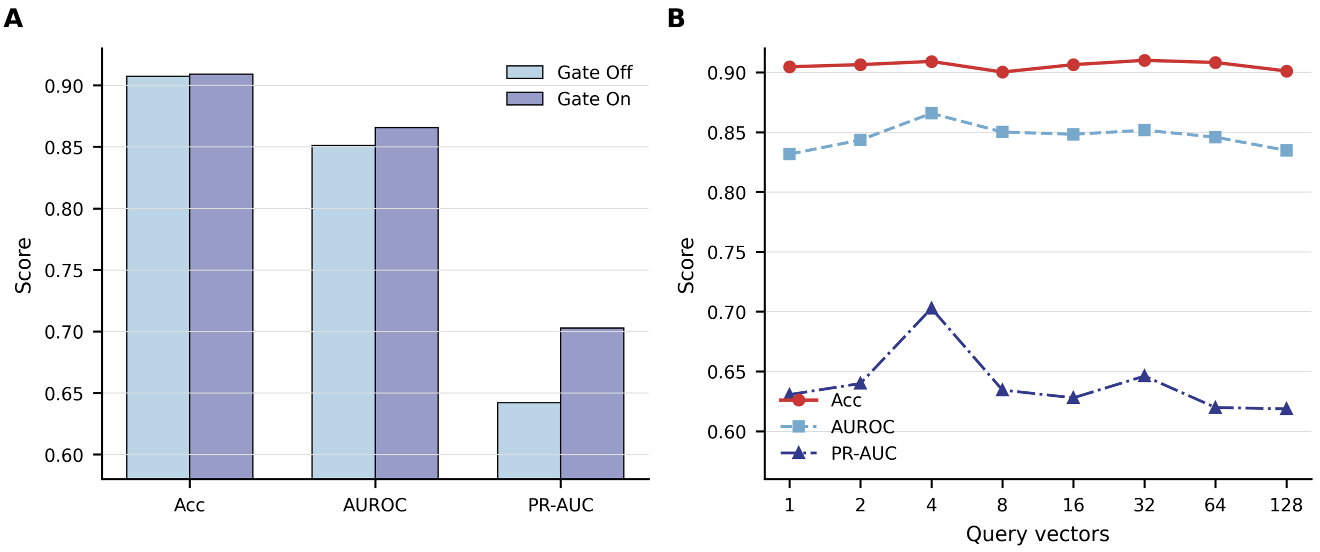

Figure S5: Ablation of Gated Query Pooling (GQP). **(A)** Performance with the gating mechanism turned off versus on. Enabling the gate yields slightly higher accuracy and AUROC and a larger improvement in PR-AUC. **(B)** Effect of the number of query vectors P on downstream performance. Accuracy, AUROC, and PR-AUC are relatively insensitive to P, with PR-AUC peaking around $P = 4$. We therefore use $P = 4$ as the default number of query vectors in all experiments.

S3 Attention-based Diagnostics

Gated Query Pooling (GQP) yields an explicit final attention tensor $\mathbf{A}^{\mathrm{g}} \in \mathbb{R}^{B\times P\times L}$ after masking, gating, and renormalization (Algorithm 1). To enable residue-level diagnostics, we record token-wise attention distributions for each query together with the query-gated readout allocation pattern for every input. We interpret these quantities conservatively as routing signals rather than definitive explanations, since attention may be an unreliable proxy for feature importance [8, 9].

Per-query attention. For a single peptide, let m denote the number of queries ($m = P$), and let $\tilde{\mathbf{A}} \in \mathbb{R}^{m\times L}$ denote the post-gating attention matrix, where $\tilde{A}_{p\ell}$ is the gated-and-renormalized attention weight assigned by query p to token position ℓ. Padding positions, if present, are masked following the same convention as GQP (i.e., logits are masked prior to the softmax operation), so that attention is normalized over valid tokens:

$$\sum_{\ell\in\mathcal{V}} \tilde{A}_{p\ell} = 1, \qquad \forall p \in \{1,\dots,m\},$$

where $\mathcal{V}$ denotes the set of valid (non-padding) token indices. In practice, $\mathcal{V}$ is constructed from tokenizer-provided masks by retaining tokens that are both attended and not marked as special tokens. For prompt pooling with head-wise gating, the internal gated attention is $\tilde{A}^{(h)}_{p\ell}$, and the exported per-query map is the head-averaged attention:

$$\tilde{A}_{p\ell} = \frac{1}{H}\sum_{h=1}^{H} \tilde{A}^{(h)}_{p\ell},$$

which reduces to the same expression when $H = 1$.

Gated attention mass. To summarize how readout evidence is allocated after gating, we define the token-level gated attention mass from $\tilde{\mathbf{A}}$ as

$$M_\ell = \frac{1}{m}\sum_{p=1}^{m} \tilde{A}_{p\ell}.$$

Thus, M_ℓ increases when multiple gated queries place probability mass on the same token position. If padding exists, we set $M_\ell = 0$ for masked positions.

Amino acid-level class contrast. For each task and class label $y \in \{0, 1\}$, we aggregate token-level masses into a frequency-weighted amino acid summary. In this aggregation, we exclude padding and special tokens (e.g., CLS/SEP/EOS) and retain only residue positions that map to the 20 canonical amino acids. Let $x_{n\ell}$ denote the amino acid identity at position ℓ in sequence n, and let $\mathcal{R}_n$ denote the set of valid residue positions for that sequence. We compute

$$M_y(\mathrm{aa}) = \frac{1}{N_y} \sum_{n:y_n=y} \sum_{\ell \in \mathcal{R}_n} M_{n\ell}\, \mathbf{1}[x_{n\ell} = \mathrm{aa}], \tag{S3.1}$$

where N_y is the total number of valid residue positions in class y. Operationally, this corresponds to summing per-residue attention mass for each amino acid within a class and normalizing by the total count of valid residues in that class. The reported class contrast is

$$\Delta M(\mathrm{aa}) = M_1(\mathrm{aa}) - M_0(\mathrm{aa}). \tag{S3.2}$$

Positive values indicate residues receiving higher gated attention mass in the positive class.

Faithfulness check via attention-guided token deletion. To test whether attention mass is nontrivial as a diagnostic signal, we perform an attention-guided deletion experiment and compare it to random deletion. For each sequence, we rank amino-acid tokens by aggregate saliency s_{bl} and progressively delete the top-ranked fraction of residues by masking them from the input. We then recompute predictions and report the relative drop in held-out accuracy as a function of the intervention fraction. As a baseline, we repeat the same procedure with uniformly random deletion of the same number of residues. Figure S6 shows that removing high-attention residues produces substantially larger accuracy drops than random deletion across all three tasks, supporting that GQP attention concentrates on task-relevant evidence rather than behaving as a trivial or arbitrary distribution. Across all three tasks, high-saliency deletion causes substantially larger accuracy drops than random deletion, and the separation increases with the intervention fraction. This consistent gap supports that GQP attention mass is nontrivial and preferentially concentrates on residues that are important for prediction.

Independent occlusion and secondary-structure robustness checks for interpretation. To further assess whether GQP attention weights agree with an attention-independent importance measure, we performed a residue-level occlusion cross-check. For each evaluated residue, we replaced the residue with `X`, recomputed the model output, and used the absolute logit change as a perturbation-based importance score. We then compared residue-level occlusion importance with GQP attention mass using Spearman rank correlation and top-k overlap. Across correctly predicted validation examples in the robust evaluation set ($n = 686$; 229 hemolysis, 243 non-fouling, and 214 solubility peptides), aggregate agreement was not consistently high; for example, mean top-k overlaps were 0.2200, 0.2250, and 0.2346 for hemolysis, non-fouling, and solubility, respectively. These results indicate that attention should not be interpreted as a globally faithful causal attribution method. Instead, we treat attention as a model-internal diagnostic routing signal and rely on controlled substitution effects (CSE) for actionable residue-edit interpretation. At the same time, selected local cases showed substantial agreement between attention and occlusion. For example, in the hemolysis validation peptide `AARIILRDRFR`, attention and occlusion agreed on residues 7R and 9R, with Spearman correlation 0.718 and top-k overlap 0.667 (Fig. S7, panel a).

To address the possibility that CSE rankings are confounded by secondary-structure propensity, we repeated the CSE ranking analysis after adding helix, sheet, turn, or combined helix/sheet/turn propensity controls to the original stratification variables (sequence length, net charge, and hydrophobic fraction). The solubility

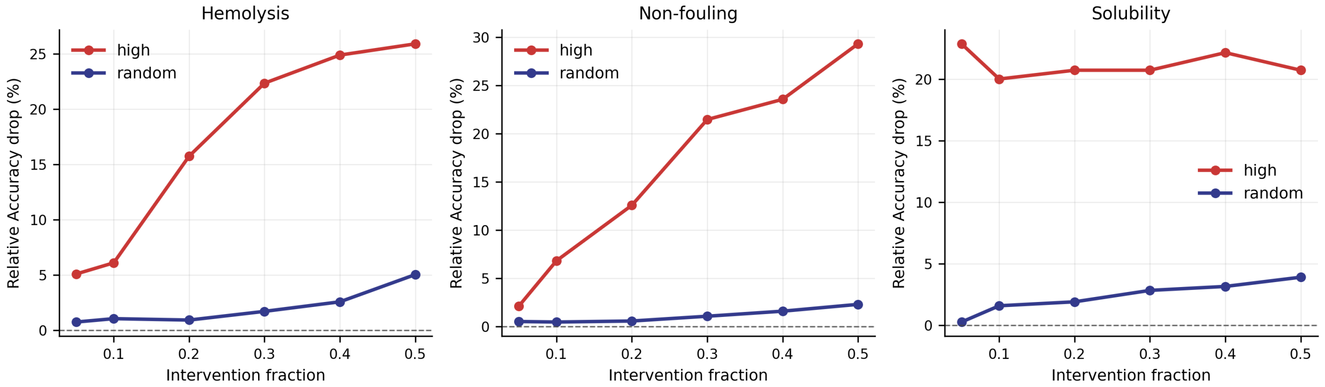

Figure S6: Attention-guided token deletion yields larger performance drops than random deletion. Faithfulness check comparing deletion of high-saliency residues (red) against random deletion (blue) on held-out test sets for hemolysis, non-fouling, and solubility. For an intervention fraction α, we delete the top-α fraction of residues ranked by aggregate saliency s_{bl} and measure the relative accuracy drop, then compare to deleting the same fraction of residues uniformly at random. Higher drops under high-saliency deletion indicate that attention mass is nontrivial and aligned with model-relevant evidence.

ranking was unchanged under all secondary-structure controls (Top-5 overlap = 1.0). The non-fouling ranking retained high Top-5 overlap (0.8–1.0), while the hemolysis ranking showed moderate sensitivity (0.6–0.8). Thus, the leading CSE residues are broadly stable to secondary-structure controls, but the rankings are not completely invariant; we therefore interpret CSE as a controlled perturbation diagnostic rather than a definitive structural mechanism.

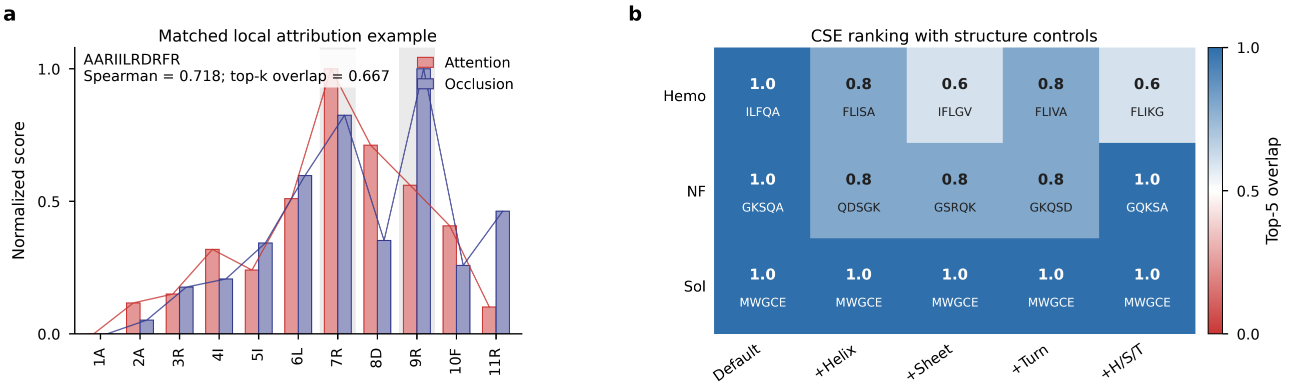

Figure S7: Independent attribution and secondary-structure robustness checks for interpretation. a, Representative hemolysis validation peptide AARIILRDRFR showing local agreement between GQP attention mass and residue-level occlusion importance. Highlighted positions indicate overlapping top-ranked residues between the two methods. **b**, Top-5 overlap between the default CSE-based residue ranking and rankings recomputed with additional secondary-structure propensity controls. Numbers denote Top-5 overlap, and amino-acid strings denote the corresponding Top-5 ranked residues under each control scheme.

```
def CSE(seqs, score_fn, AA20, stratum_id, T=1.0):
    # U/C: (K,20,20), from-AA -> to-AA
    U_sum, U_cnt = init_tables()
    S_sum, S_cnt = init_stratum_tables()   # keyed by (s,f,t)
    W_cnt = init_row_weight_counts()       # keyed by (f,s)

    for n, x in enumerate(seqs):
        base = score_fn([x])[0]            # (K,)
        s = stratum_id[n]
        for i, aa_from in enumerate(x):
            if aa_from not in AA20: continue
            f = AA20.index(aa_from)
            W_cnt[f, s] += 1
            for t, aa_to in enumerate(AA20):
                if t == f: continue
                x_mut = x[:i] + aa_to + x[i+1:]
                d = (score_fn([x_mut])[0] - base) / max(T, 1e-3)  # delta-logit
                U_sum[:, f, t] += d; U_cnt[f, t] += 1
                S_sum[s, f, t] += d; S_cnt[s, f, t] += 1

    U = safe_mean(U_sum, U_cnt)            # uncontrolled
    C = zeros_like(U) * nan                # controlled (stratified reweight)
    for f in range(20):
        w = normalize_over_strata(W_cnt[f, :])      # row weights for from-AA=f
        for t in range(20):
            if t == f: continue
            C[:, f, t] = sum_s w[s] * safe_mean(S_sum[s, f, t], S_cnt[s, f, t])

    interv = row_mean_offdiag(C)       # (K,20)
    return U, C, interv
```

Listing 2: CSE (very minimal pseudocode).

S4 Controlled Substitution Effects

Controlled Substitution Effects (CSE) is a perturbation-based diagnostic procedure that quantifies how model predictions change under single-residue edits of an input peptide sequence. The method is aligned with in silico saturation mutagenesis (ISM), which evaluates one-site substitutions and records output shifts [10, 11]. In our implementation, "controlled" does not mean restricting the substitution alphabet at each site. Instead, we first compute full ISM effects and then apply sequence-level stratified reweighting to control for coarse confounders.

Setup and notation. Let $x = (x_1, \ldots, x_L)$ be an amino-acid sequence of length L, and let $f(x)$ denote the diagnostic score (in our implementation, logit-scale score; for binary tasks, pre-sigmoid logit). For a single substitution at position i, the counterfactual effect is

$$\Delta(i \to a) = \frac{f\left(x^{(i\to a)}\right) - f(x)}{\max(T, 10^{-3})}, \tag{S4.1}$$

where T is the calibration temperature (default $T = 1$ if unavailable). This corresponds to ISM-style single-residue scans [12, 13].

Uncontrolled scan. For each sequence, CSE evaluates all valid positions and all substitutions in $\mathscr{A} \setminus \{x_i\}$ (no per-position candidate filtering). Effects are aggregated into an uncontrolled transition table indexed by from-amino-acid and to-amino-acid, $U_{k,ab}$ for $a, b \in \mathscr{A}$ with $a \neq b$, where k indexes task/logit.

Sequence-level confounder control. To control for coarse distributional differences across sequences, we stratify source sequences by sequence-level confounders (L, Q, H), where L is sequence length, Q is net charge (with configurable Histidine weight), and H is hydrophobic fraction. Controlled effects are then computed via stratified reweighting (g-formula style) within each from-AA row, yielding $C_{k,ab}$. In code, this corresponds to the `control=stratify` setting.

Position handling. By default, all valid positions are scanned. When a from-amino-acid appears multiple times in one sequence, the implementation supports within-sequence averaging (default) and random-occurrence mode with repeats (for uncertainty diagnostics).

Aggregated CSE scores. We report both uncontrolled (U) and controlled (C) transition effects, and define residue-level intervenability as

$$\text{Intervenability}_k(a) = \frac{1}{|\mathscr{A}| - 1} \sum_{b \neq a} C_{k,ab}. \tag{S4.2}$$

Higher intervenability indicates that editing from residue a tends to induce larger model-score changes after confounder control.

A simplified implementation is shown above. The procedure is model-agnostic and can be applied to any sequence-to-score function. In this work, attention and CSE are treated as complementary diagnostics; CSE effect sizes are always defined by counterfactual logit differences in Eq. S4.1.

Sensitivity of CSE-based residue prioritization to stratification choices. To assess how residue-level prioritization depends on the stratified control used to compute controlled substitution effects (CSE; Δlogit), we recompute the per-residue intervenability rankings under alternative stratification settings and compare them to the default ranking. We quantify agreement using the Top-5 overlap between the default intervenability ranking and the ranking obtained under each alternative setting. Figure S8 summarizes robustness across tasks for variations in binning granularity, removing length binning, and alternative hydrophobicity and charge definitions. Overall, the Top-5 overlap is moderate-to-high across most alternative settings, indicating that the highest-priority residues are largely stable to reasonable changes in binning granularity and the inclusion of length. The largest shifts occur under specific alternative physicochemical definitions, suggesting that residue prioritization is more sensitive to how hydrophobicity and charge are defined than to modest changes in bin width. We therefore treat these alternative definitions as a useful stress test, while using the default stratification for the main intervenability summaries.

S5 Attention Visualization Examples

This section provides qualitative attention visualizations to complement the quantitative analyses in the main text (Figure 3) and the diagnostic methodology described in Sections S2 and S3. Specifically, we show representative token-level heatmaps derived from the Gated Query Pooling (GQP) readout for the hemolysis classifier, with examples from both **no-hemolysis** and **hemolysis** classes. The purpose is to illustrate how the model routes evidence across residues in individual sequences and to provide concrete sanity checks for the aggregate trends reported in the main text, rather than to claim attention as a definitive causal explanation.

For each peptide, we visualize a per-token diagnostic weight obtained from the GQP attention map returned by the pooling operator. In practice, we aggregate attention across the P learnable queries (and, when gating

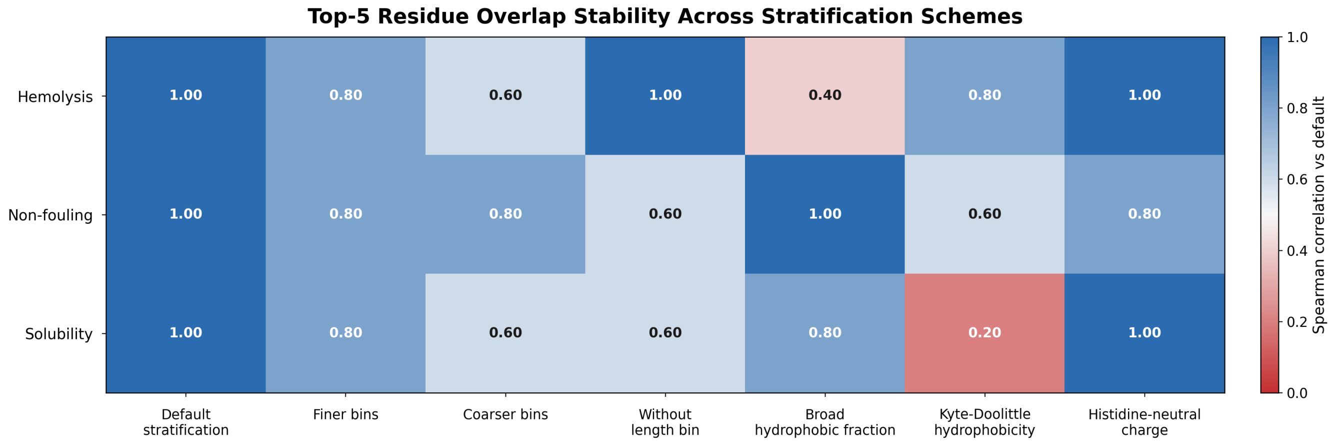

Figure S8: Robustness of Top-5 residue prioritization under alternative stratification settings. Heatmap of Top-5 overlap (range 0–1) between the default CSE-based intervenability ranking and rankings recomputed with alternative confounder controls. Rows correspond to tasks (hemolysis, non-fouling, solubility), and columns correspond to stratification schemes, including finer/coarser binning, removal of length binning, and alternative hydrophobicity and charge definitions. Higher values indicate greater agreement with the default ranking, whereas lower values indicate stronger sensitivity to the stratification choice.

is enabled, optionally incorporate the gate-modulated contribution) to obtain a single scalar weight per token. Special tokens (<cls> and <eos>) are displayed for completeness, but interpretation focuses on amino-acid tokens; masking rules follow the same convention as in the implementation details (i.e., padding/special-token handling consistent with Section S2). Warmer colors indicate tokens receiving higher evidence weight under the GQP readout.

Figure S9 shows three representative sequences per class. Across examples, the diagnostic weights are non-uniform and sequence-dependent, with localized high-weight segments rather than diffuse, uniform patterns. This qualitative behavior is consistent with the intent of GQP: different learnable queries attend to different subsets of residues, and their merged representation emphasizes tokens that are most informative under the trained classifier. Importantly, we treat these maps as diagnostic routing signals rather than stand-alone explanations. In particular, attention distributions can vary without substantially changing model outputs, and they may not align with perturbation-based importance in all cases. Therefore, whenever we translate diagnostic signals into actionable edit hypotheses, we rely on controlled counterfactual substitutions to quantify prediction sensitivity under minimal, single-residue edits.

S6 Residue-level Intervenability for PE, PP, and PET

Figure S10 provides a residue-centric complement to the position-level intervention analysis in the main text (Fig. 6) and the CCS-based diagnostics described in Section S4. Specifically, for each plastic target—**PE** (panel A), **PP** (panel B), and **PET** (panel C)—we aggregate controlled counterfactual substitution effects by the introduced amino-acid identity and report the resulting mean intervenability (mean MSE) as a ranked bar plot.

For PE, intervenability is sharply dominated by `W`, followed by `Y` and then a smaller set of residues (`R`, `F`, `K`, `L`, `M`, `H`, `E`) with markedly lower mean effects. Most remaining residues have near-zero intervenability, indicating that introducing them under controlled single-site edits produces little change in the predicted PE-binding score on average. This pattern is consistent with the strong enrichment of aromatic residues observed after

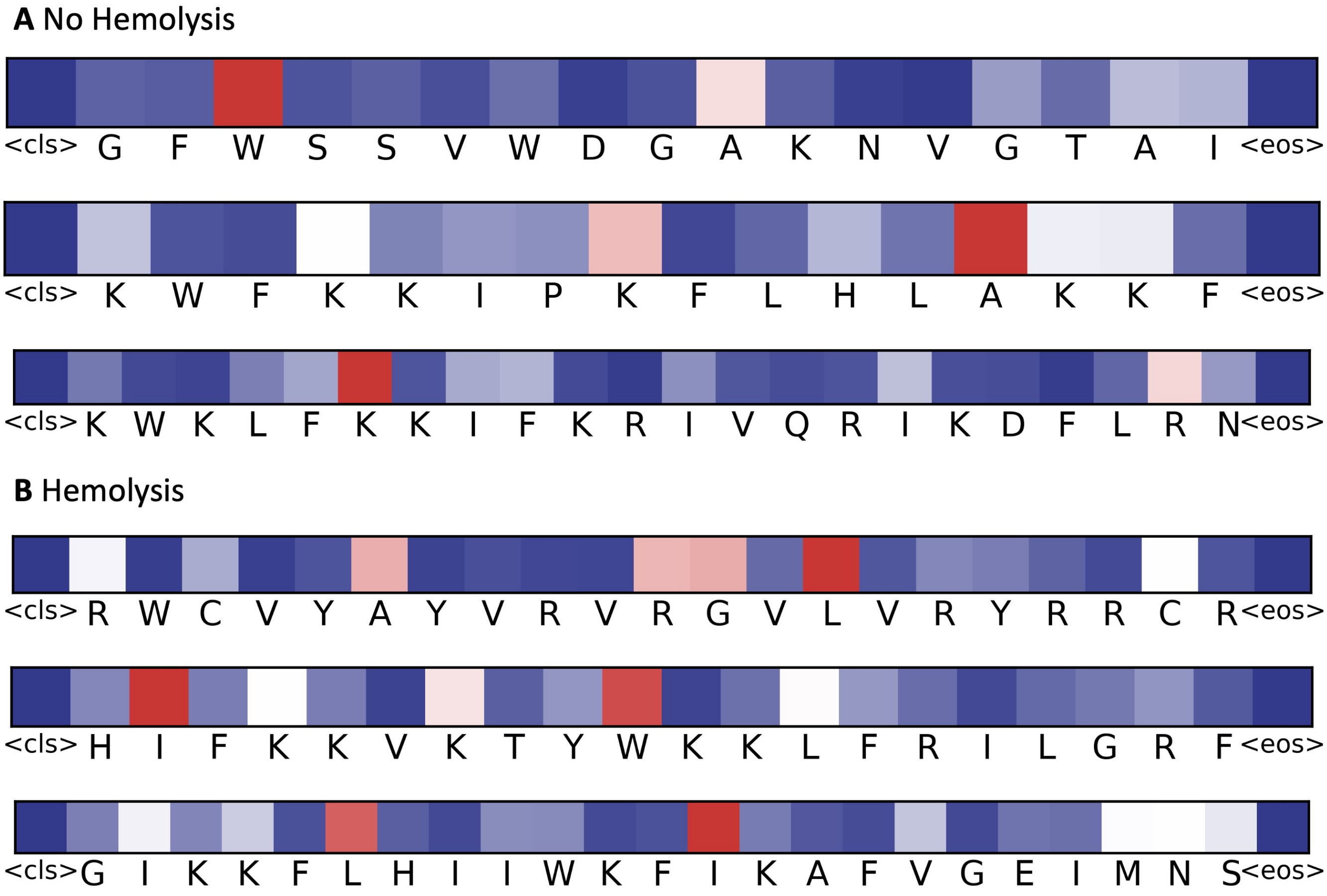

Figure S9: Representative attention visualizations for hemolysis prediction. Additional examples complementing Figure 3, showing token-level GQP diagnostic weights for **no-hemolysis** (A) and **hemolysis** (B) peptides. Colors indicate the relative evidence weight assigned to each token under the GQP readout (including <cls> and <eos>), aggregated across queries for visualization.

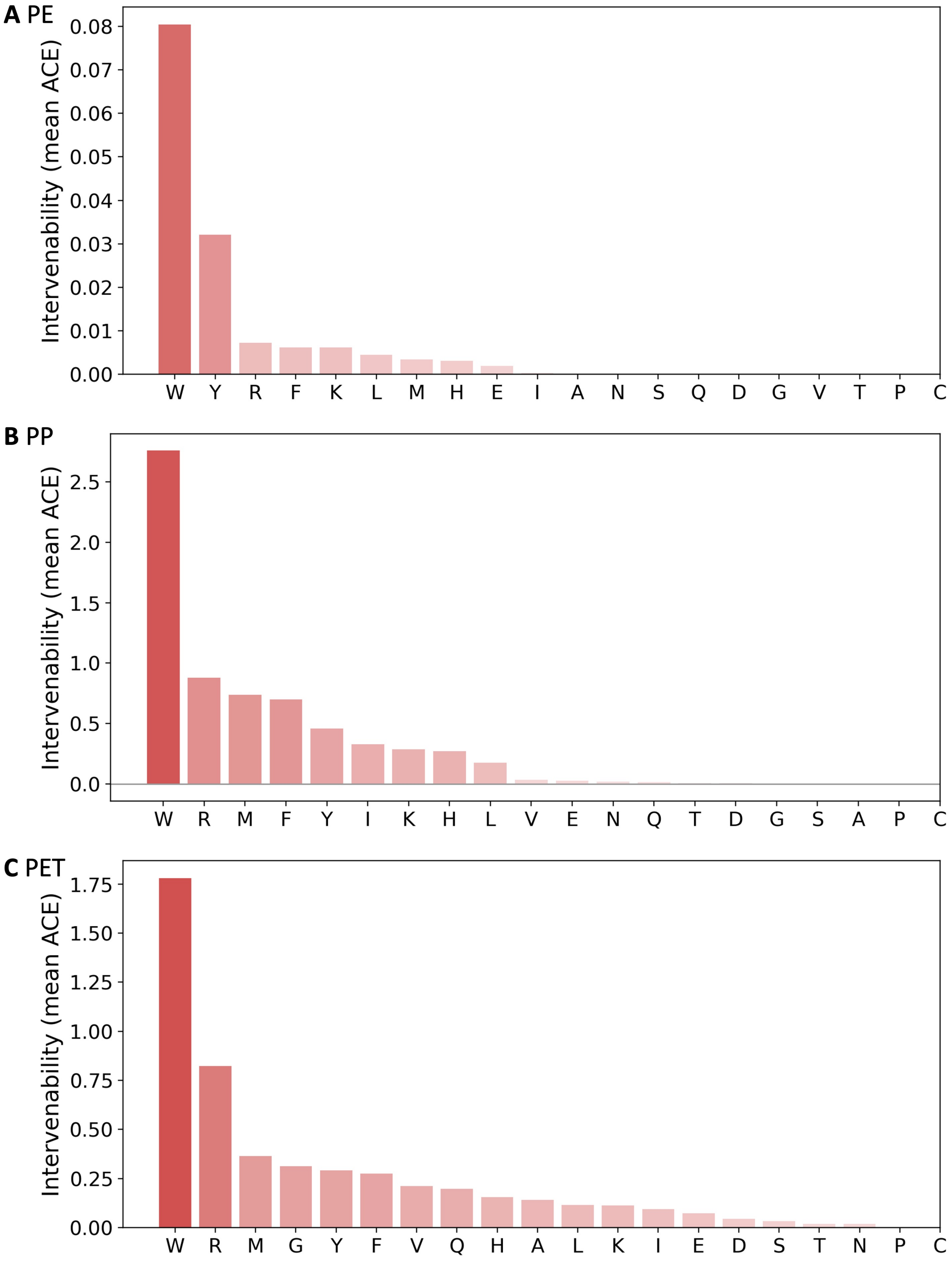

Figure S10: Residue-level intervenability profiles for PE, PP, and PET. Mean intervenability (mean CSE; computed from controlled counterfactual substitutions, Section S4) is shown as a ranked bar plot over amino-acid identities for (A) PE, (B) PP, and (C) PET. Bars are sorted within each panel from highest to lowest, indicating which residue types most strongly perturb the target-specific binding score under minimal, controlled single-site edits.

the second-stage screening (see the compositional heatmaps and the final PE candidates in Table S2).

For PP, `W` again ranks as the most intervenable residue, with a broader second tier (`R`, `M`, `F`, `Y`, `I`, `K`, `H`, `L`) showing substantial but smaller effects. Compared with PE, the PP profile is less concentrated, suggesting that multiple residue identities can act as effective levers for score modulation under the same controlled substitution protocol. Residues in the tail (e.g., `E`, `N`, `Q`, `T`, `D`, `G`, `S`, `A`, `P`, `C`) contribute minimally.

For PET, `W` and `R` dominate, followed by a moderate tier including `M`, `G`, `Y`, `F`, and `V`. The remaining residues show progressively smaller effects. Notably, the PET panel reflects the most stringent final-stage selection in our pipeline (only one PET sequence retained; Table S2), and the intervenability profile highlights which residue identities most strongly perturb the PET score under controlled edits.

Figure 6 addresses where edits matter most (position-level sensitivity), whereas Figure S10 summarizes which residue identities tend to be the most effective single-site interventions for each target (PE/PP/PET). Because the absolute scale of mean CSE can vary across targets due to differences in score distributions and candidate pools, Figure S10 is most informative for within-target comparisons (i.e., ranking residue types for PE, PP, or PET separately) rather than direct cross-target magnitude comparisons.

S7 Final Selected Peptide Candidates

Interpretation of sequence-pattern shifts across filtering stages. Figure S11 summarizes how the two-stage screening pipeline (main text Fig. 5–6) reshapes position-wise residue usage for each plastic target. Across all three targets, the `before` and `after-1st` panels exhibit broadly diffuse residue distributions with similar qualitative patterns, indicating that the first-stage filter substantially reduces the pool size while preserving relatively diverse positional composition. By contrast, the `after-2nd` panels become highly structured and sparse (with $n = 5$ for `pe`, $n = 5$ for `pp`, and $n = 1$ for `pet`), reflecting strong convergence toward a small set of position-specific motifs.

This convergence is also consistent with the final sequences (Table S2), which are enriched in aromatic and basic residues (notably `W/H/R`) and display recurring local patterns (e.g., clustered `W` and `H` segments) aligned with the concentrated blocks observed in the `after-2nd` heatmaps. Importantly, the sparsity in the final-stage maps should not be interpreted as evidence that any single residue position is causally responsible for binding. Rather, it indicates that, under the adopted objective and screening criteria, only a small subset of motifs remains competitive. Accordingly, we treat positional enrichment as a diagnostic signature of selection pressure and complement it with mechanistic sanity checks using attention-based diagnostics and controlled counterfactual substitutions, which quantify whether model scores are sensitive to minimal single-residue edits at highlighted positions.

Here we report the final peptide candidates retained after secondary screening on the PepBD-derived microplastic adsorption-scoring dataset (see Section S1 for data provenance and screening protocol). We list the target plastic substrate (PE/PP/PET), the within-target rank, and the PepBD score as provided by the source dataset. Scores are reported as-is (more negative indicates stronger binding under the PepBD scoring convention used in the source), and ranks are computed within each target by sorting candidates by score.

PepBD threshold sensitivity analysis. To test how the final microplastic-binding candidates depend on the plastic-specific PepBD score cutoffs used in the main screening workflow, we performed a threshold-sensitivity analysis while keeping the developability filters fixed at hemolysis probability < 0.5, non-fouling probability ≥ 0.5, and solubility probability ≥ 0.5. The baseline PepBD score thresholds were PE ≤ -56, PP ≤ -50, and PET ≤ -60, which reproduce the 11 final hits reported in the main text (5 PE, 5 PP, and 1

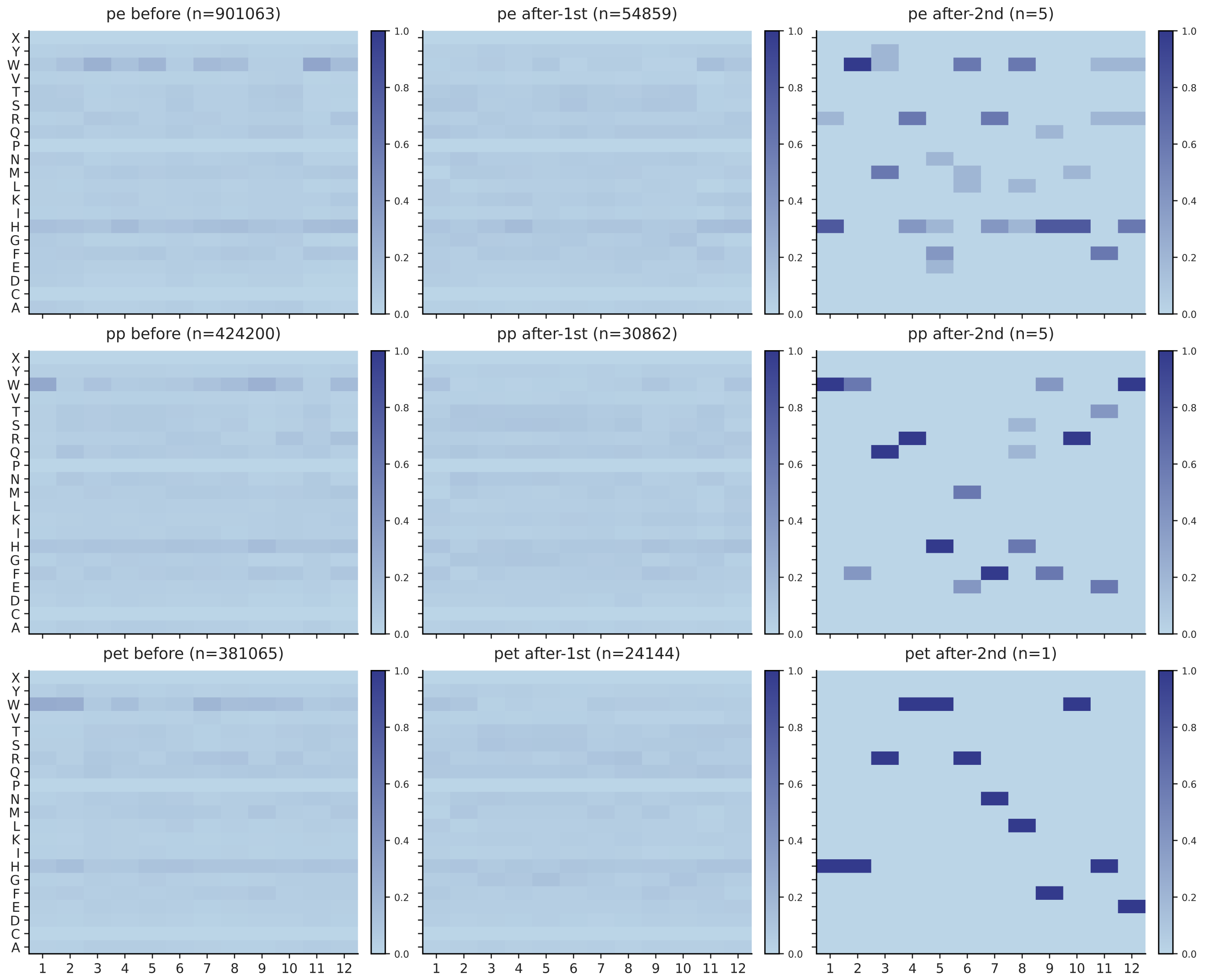

Figure S11: Position-wise amino-acid usage across the screening pipeline. For each target plastic (pe, pp, pet), heatmaps report residue frequencies at positions 1–12 before screening, after the first-stage filter, and after the second-stage filter (main text Fig. 5–6); n denotes the number of sequences retained at each stage. Colors indicate normalized frequencies (darker = higher), illustrating how successive filtering concentrates the pools into a small set of motifs, consistent with the final candidates in Table S2.

PET). We then shifted all three PepBD thresholds together by -2, -1, $+1$, $+2$, and $+5$ score units, and also varied one plastic-specific threshold at a time by -1, 0, and $+1$ score units. Under a modest global relaxation of $+1$ or $+2$ score units, all 11 baseline hits were retained and 6 or 13 additional candidates were added, respectively. Under a modest global tightening of -1 score unit, 7 of the 11 baseline hits were retained. When one plastic-specific threshold was varied at a time, PE and PP each lost two target-specific hits under a -1 shift and gained 5 and 1 hits under a $+1$ shift, respectively, whereas PET remained at one hit from -1 to $+1$. Thus, the exact number of final candidates is threshold-dependent, as expected for an extreme-tail screen, while the reported candidates form a conservative subset that is retained under modest relaxation of the PepBD thresholds.

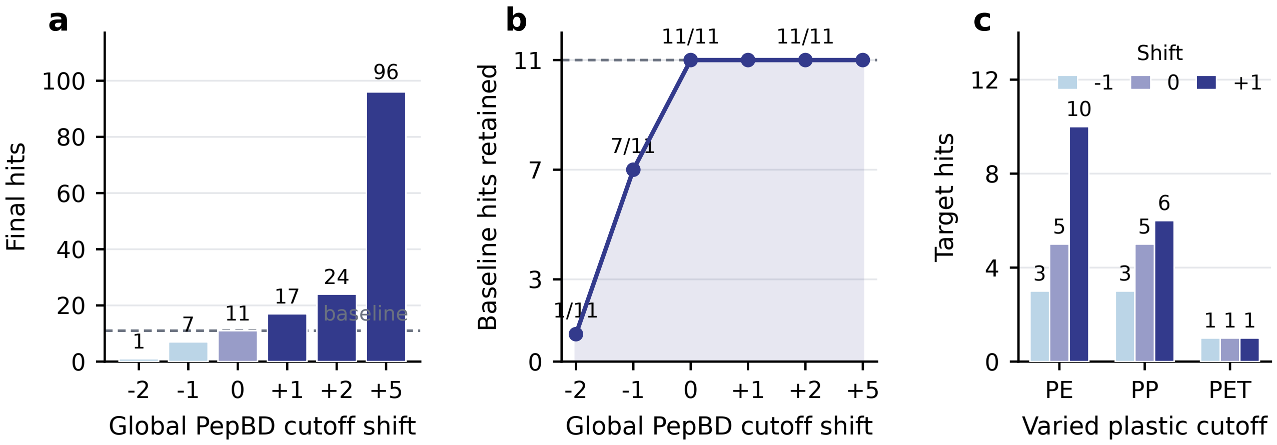

Figure S12: Sensitivity of final microplastic-binding candidates to PepBD score thresholds. **a**, Total number of final hits when all three plastic-specific PepBD score thresholds are shifted together relative to the baseline thresholds (PE ≤ -56, PP ≤ -50, PET ≤ -60). **b**, Number of the 11 baseline hits retained under the same global threshold shifts. **c**, Target-specific hit counts when one plastic-specific threshold is varied by -1, 0, or $+1$ score unit while the other two PepBD thresholds and all developability filters are fixed. Negative shifts are stricter cutoffs and positive shifts are more permissive cutoffs.

Simulation Setup

To complement sequence-level screening with physics-based triage, we established a unified coarse-grained molecular dynamics (MD) framework using Martini 3 with GPU-accelerated GROMACS. All candidates were evaluated under identical thermodynamic conditions (310 K, 0.15 M NaCl, Martini water) and a shared integration protocol, enabling direct comparability across peptides and across proxy tasks.

The validation campaign comprised three experiment families: membrane interaction (hemolysis proxy), multi-copy self-association in bulk water (solubility proxy), and surface adsorption tendency (non-fouling proxy). The final completed dataset includes 13 sequences (11 screened candidates + 2 controls), each with three independent replicates per proxy (39 trajectories per proxy family; 117 production trajectories in total). Analysis metrics and thresholds were defined a priori and applied uniformly to all sequences, minimizing post hoc tuning and improving reproducibility of cross-candidate ranking.

Sampling-time limitation. The present Martini 3 simulations are intended as short-timescale, relative physical triage rather than exhaustive sampling of peptide–environment states. Although coarse-grained

Table S2: Final selected peptide candidates after secondary screening. Candidates are grouped by target plastic (PE/PP/PET) and ranked within each target by PepBD score.

Target	Rank	Sequence	PepBD score
**PE**			
PE	1	HWMRFWRWHHFH	-57.49
PE	2	HWMREWRWHHFH	-57.34
PE	3	HWWHHMHLQHWR	-57.10
PE	4	HWMRNWRWHHFH	-56.95
PE	5	RWYHFLHHHMRW	-56.40
**PP**			
PP	1	WWQRHMFHFREW	-51.82
PP	2	WWQRHMFSFREW	-51.39
PP	3	WFQRHMFHWREW	-51.13
PP	4	WFQRHEFHWRTW	-50.54
PP	5	WWQRHEFQFRTW	-50.12
**PET**			
PET	1	HHRWWRNLFWHE	-62.62

simulations accelerate diffusive motions relative to all-atom simulations, the 0.5–1.0 μs production windows used here may still be insufficient to observe slow peptide aggregation, membrane insertion, or surface-adsorption events, particularly if such events require rare orientations, multi-step rearrangements, or longer nucleation times. Therefore, the absence of persistent membrane contact, stable aggregation, or surface adsorption in these trajectories should be interpreted as "not detected under the present protocol" rather than as evidence that these events cannot occur. Longer simulations, multiple initial configurations, enhanced-sampling strategies, and experimental assays will be needed before making absolute safety, solubility, or anti-fouling claims for individual candidates.

Hemolysis Proxy

To assess membrane-disruption liability at the screening stage, we performed coarse-grained membrane simulations as a hemolysis-risk proxy and quantified three readouts: membrane contact fraction, peptide insertion depth relative to the bilayer center, and water-defect fraction in the membrane core. The primary hemolysis endpoint used for ranking in this appendix is insertion depth (Fig. S13, panel A). Across the final completed set (11 candidates + 2 controls; 3 replicates each), membrane contact fraction remained uniformly zero in all trajectories, indicating no persistent membrane-bound state within the present sampling window. Insertion depth varied across candidates (mean range: 13.30–18.10 nm), whereas controls spanned a wider range (CTRL-POS: 12.59 nm; CTRL-NEG: 21.78 nm). Water-defect fractions were uniformly low for all sequences (candidate means: 0.002–0.027), and no candidate exhibited sustained high-defect behavior under this setup. Collectively, these trajectories do not support strong membrane-active behavior for the screened candidates under the present protocol. We therefore interpret the hemolysis MD analysis as low-risk relative triage evidence within this candidate panel, while avoiding direct claims about absolute hemolysis endpoints.

Table S3: Simulation protocol for MD-based physical triage. A single, fixed setup was used for all peptides to enable unbiased cross-candidate comparison.

Category	Configuration
Simulation framework	Martini 3 coarse-grained force field; GPU-accelerated GROMACS; standard coarse-grained peptide and system builders
Global state variables	Temperature = 310 K; ionic strength = 0.15 M NaCl; Martini water model
Replicate design	3 independent replicates per peptide for each proxy experiment
Integrator and stabilization	Time step = 10 fs; sequential energy minimization and equilibration (NVT/NPT) before production; consistent trajectory/energy/log output intervals across all systems
Hemolysis proxy (Experiment A)	Single peptide above a POPC:CHOL (70:30) bilayer; initial peptide–membrane separation = 3.5 nm; box = $12 \times 12 \times 16$ nm; production = 1.0 μs per replicate
Solubility proxy (Experiment B)	12 peptide copies in bulk water; box = $16 \times 16 \times 16$ nm; production = 1.0 μs per replicate
Non-fouling proxy (Experiment C)	Single peptide near a generic hydrophobic surface potential; box = $12 \times 12 \times 18$ nm; production = 0.5 μs per replicate
Primary analysis thresholds	Membrane-contact cutoff = 0.6 nm; water-defect core half-thickness = 1.0 nm; clustering cutoff = 0.65 nm; aggregation threshold = cluster size ≥ 6; surface-contact cutoff = 0.6 nm

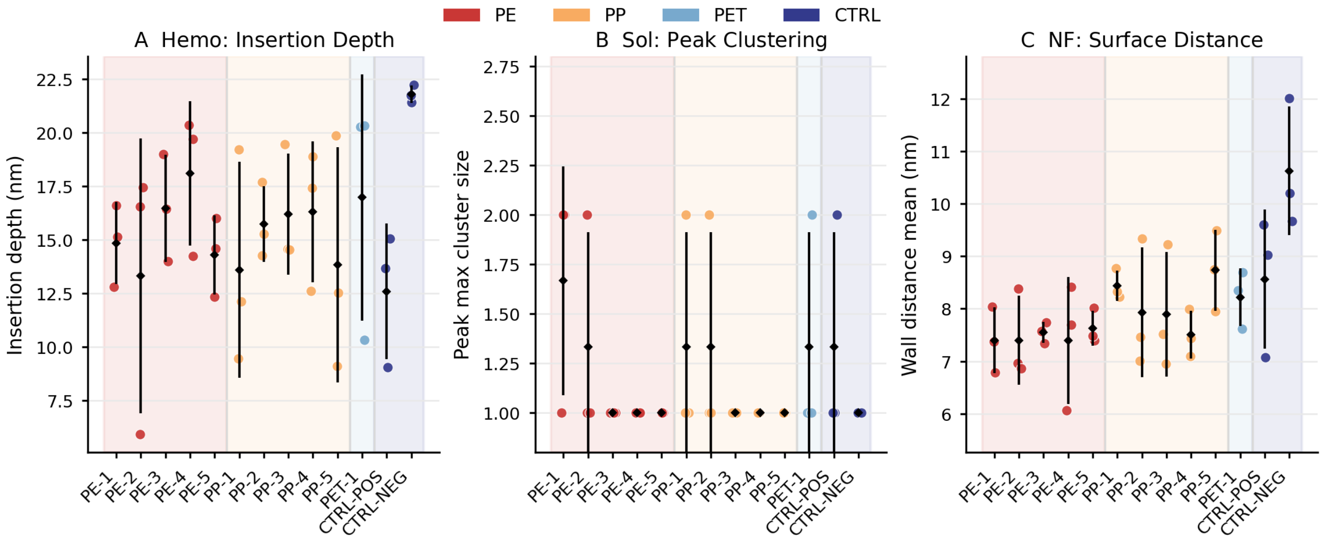

Figure S13: Primary MD proxy endpoints used for final triage. Integrated summary across all completed runs (11 candidates + 2 controls; 3/3 replicates each): **A**, hemolysis proxy (insertion depth); **B**, solubility proxy (peak cluster size); **C**, non-fouling proxy (mean wall distance). Points denote replicate values; black diamonds and error bars indicate mean and standard deviation across replicates.

Solubility Proxy

To evaluate aggregation liability as a proxy for solution-phase behavior, we analyzed multi-copy coarse-grained simulations (Experiment B) using cluster-based readouts: maximum cluster size, mean cluster size,

Table S4: Hemolysis-proxy summary for peptides with completed hemo simulations (3 replicates each). Values are reported as mean ± SD across replicates.

Peptide	Replicates	Contact fraction	Insertion depth (nm)	Water-defect fraction
PE-1	3	0.000 ± 0.000	14.84 ± 1.92	0.015 ± 0.008
PE-2	3	0.000 ± 0.000	13.30 ± 6.41	0.027 ± 0.042
PE-3	3	0.000 ± 0.000	16.47 ± 2.50	0.014 ± 0.020
PE-4	3	0.000 ± 0.000	18.10 ± 3.37	0.002 ± 0.002
PE-5	3	0.000 ± 0.000	14.30 ± 1.85	0.003 ± 0.005
PP-1	3	0.000 ± 0.000	13.59 ± 5.04	0.020 ± 0.030
PP-2	3	0.000 ± 0.000	15.73 ± 1.76	0.012 ± 0.020
PP-3	3	0.000 ± 0.000	16.18 ± 2.82	0.009 ± 0.009
PP-4	3	0.000 ± 0.000	16.30 ± 3.28	0.008 ± 0.012
PP-5	3	0.000 ± 0.000	13.83 ± 5.50	0.019 ± 0.025
PET-1	3	0.000 ± 0.000	16.97 ± 5.75	0.019 ± 0.014
CTRL-POS	3	0.000 ± 0.000	12.59 ± 3.15	0.008 ± 0.010
CTRL-NEG	3	0.000 ± 0.000	21.78 ± 0.41	0.005 ± 0.004

and aggregation-onset time. The primary solubility endpoint used for ranking is peak cluster size (Fig. S13, panel B). Across the final completed set (11 candidates + 2 controls; 3 replicates each), no aggregation-onset event was detected in any replicate within the sampled window. Most sequences remained fully monomeric (peak maximum cluster size = 1). A subset exhibited only transient dimer-like excursions (peak = 2 in at least one replicate), including PE-1, PE-2, PP-1, PP-2, PET-1, and CTRL-POS. Mean cluster-size readouts remained effectively 1.0 for all sequences, indicating no stable multi-copy aggregation. Overall, these results support low aggregation propensity in this MD proxy setting and are interpreted as relative solubility triage rather than absolute solubility quantification.

Non-fouling Proxy

To quantify nonspecific adsorption tendency, we analyzed Experiment C trajectories using three readouts: surface-contact fraction, adsorption-event count, and mean wall distance. The primary non-fouling (NF) endpoint used for ranking is mean wall distance (Fig. S13, panel C). Across the final completed set (11 candidates + 2 controls; 3 replicates each), surface-contact fractions were uniformly zero and no adsorption events were detected (Table S6). Consistently, adsorption-onset times and residence-time statistics were not observed in these runs. Although contact and event readouts were uniformly null, mean wall distance still provided a relative ordering among candidates. Candidate means ranged from 7.39 to 8.73 nm, with most PE/PP/PET candidates falling within 7.4–8.7 nm. Control means were 8.57 nm (CTRL-POS) and 10.63 nm (CTRL-NEG), consistent with weaker surface proximity for the negative control. As with the other MD proxies, we interpret this analysis as relative physical triage rather than a definitive anti-fouling endpoint.

Across the two-stage screening pipeline, position-wise residue usage transitions from diffuse distributions to highly concentrated motifs at the final stage (Fig. S11), yielding a compact set of high-scoring candidates for each plastic target (Table S2). The final sequences are consistently enriched in aromatic and basic residues (notably `W/H/R`) and exhibit recurring local patterns, mirroring the structured blocks observed in the `after-2nd` heatmaps. Complementing this sequence-level selection, the unified Martini 3 MD campaign provides a physics-based relative triage across three proxy tasks under fixed conditions (Table S3). Within the completed

Table S5: Solubility-proxy summary for all completed Experiment B results (3 replicates per sequence). Values are reported as mean ± SD across replicates.

Peptide	Replicates	Peak cluster size (mean±SD)	Peak max	Mean cluster size	Aggregation onset (ps)
PE-1	3	1.67 ± 0.58	2	1.0000 ± 0.0000	NA
PE-2	3	1.33 ± 0.58	2	1.0000 ± 0.0000	NA
PE-3	3	1.00 ± 0.00	1	1.0000 ± 0.0000	NA
PE-4	3	1.00 ± 0.00	1	1.0000 ± 0.0000	NA
PE-5	3	1.00 ± 0.00	1	1.0000 ± 0.0000	NA
PP-1	3	1.33 ± 0.58	2	1.0000 ± 0.0000	NA
PP-2	3	1.33 ± 0.58	2	1.0000 ± 0.0000	NA
PP-3	3	1.00 ± 0.00	1	1.0000 ± 0.0000	NA
PP-4	3	1.00 ± 0.00	1	1.0000 ± 0.0000	NA
PP-5	3	1.00 ± 0.00	1	1.0000 ± 0.0000	NA
PET-1	3	1.33 ± 0.58	2	1.0000 ± 0.0000	NA
CTRL-POS	3	1.33 ± 0.58	2	1.0000 ± 0.0000	NA
CTRL-NEG	3	1.00 ± 0.00	1	1.0000 ± 0.0000	NA

Table S6: Non-fouling-proxy summary for peptides with completed Experiment C simulations (3 replicates each). Values are reported as mean ± SD across replicates.

Peptide	Replicates	Surface-contact fraction	Adsorption events	Wall distance mean (nm)
PE-1	3	0.000 ± 0.000	0.00 ± 0.00	7.40 ± 0.63
PE-2	3	0.000 ± 0.000	0.00 ± 0.00	7.40 ± 0.85
PE-3	3	0.000 ± 0.000	0.00 ± 0.00	7.55 ± 0.20
PE-4	3	0.000 ± 0.000	0.00 ± 0.00	7.39 ± 1.21
PE-5	3	0.000 ± 0.000	0.00 ± 0.00	7.63 ± 0.33
PP-1	3	0.000 ± 0.000	0.00 ± 0.00	8.44 ± 0.29
PP-2	3	0.000 ± 0.000	0.00 ± 0.00	7.93 ± 1.24
PP-3	3	0.000 ± 0.000	0.00 ± 0.00	7.90 ± 1.18
PP-4	3	0.000 ± 0.000	0.00 ± 0.00	7.51 ± 0.45
PP-5	3	0.000 ± 0.000	0.00 ± 0.00	8.73 ± 0.77
PET-1	3	0.000 ± 0.000	0.00 ± 0.00	8.22 ± 0.55
CTRL-POS	3	0.000 ± 0.000	0.00 ± 0.00	8.57 ± 1.33
CTRL-NEG	3	0.000 ± 0.000	0.00 ± 0.00	10.63 ± 1.23

panel (11 candidates + 2 controls; 3 replicates each), we observed no persistent membrane-bound state (contact fraction = 0) and uniformly low membrane water-defect fractions, no detected aggregation-onset events in multi-copy bulk simulations (with peak cluster sizes remaining at 1–2), and no surface-contact/adsorption events in the non-fouling proxy. Accordingly, under the present sampling window and protocol, the MD proxies collectively support a low-liability profile relative to the included controls while primarily serving as a consistent ranking/triage layer rather than definitive measurements of hemolysis, solubility, or anti-fouling performance.

S8 GQP Code Availability and Usage

Open-source implementation. The Gated Query Pooling (GQP) training and analysis code has been released at `https://github.com/PEESEgroup/GQP`. The repository contains the model implementation, benchmark data files in JSONL format, single-task and batch-training entry points, and diagnostic scripts for attention and controlled substitution analyses. The following commands summarize the intended usage for reproducing the sequence-only GQP developability models.

Environment setup. After cloning the repository, users can create a Python environment and install dependencies as follows:

```
git clone https://github.com/PEESEgroup/GQP.git
cd GQP
python -m venv .venv
source .venv/bin/activate
pip install -U pip
pip install -r requirements.txt
```

Data layout. The benchmark files are provided under `datasets/jsonl/`. Each task folder contains sequence-level JSONL files such as `datasets/jsonl/hemo/train.jsonl`, `datasets/jsonl/hemo/val.jsonl`, `datasets/jsonl/nf/train.jsonl`, and `datasets/jsonl/sol/train.jsonl`. Each JSONL record stores a peptide sequence and its binary task label. The public repository also provides the `data_jsonl` link used by the batch script.

Single-task training and evaluation. For example, the hemolysis model can be trained with ESM2 and GQP using:

```
python train.py train \
  --train_json datasets/jsonl/hemo/train.jsonl \
  --val_json datasets/jsonl/hemo/val.jsonl \
  --out_dir runs/gqp_demo/hemo \
  --esm_backend hf \
  --esm_model facebook/esm2_t33_650M_UR50D \
  --pool_type prompt \
  --prompt_tokens 4 \
  --train_backbone

python train.py eval \
  --model_dir runs/gqp_demo/hemo \
  --test_json datasets/jsonl/hemo/val.jsonl
```

The same command pattern can be applied to `nf` and `sol` by replacing the task-specific JSONL paths and output directory.

Batch training. The three developability tasks can also be trained through the provided shell script:

```
bash train_gqp.sh

# optional examples
GPU_ID=0 bash train_gqp.sh
GPU_IDS="0,1,2" bash train_gqp.sh
TASKS="hemo sol nf" SEED=42 bash train_gqp.sh
```

By default, the batch script trains the ESM2-650M GQP model for `hemo`, `sol`, and `nf` using `pool_type=prompt`, `prompt_tokens=4`, focal loss, cosine warmup scheduling, and `SEED=42`; these settings can be overridden through environment variables. Outputs are written under `runs/`, including model checkpoints, configuration files, logs, and evaluation metrics.

Diagnostic analyses. The repository also includes scripts for reproducing the interpretation analyses. For example, attention diagnostics and controlled substitution effects can be generated with:

```
python scripts/diagnostics/attention/eval_attn_char_stats.py \
  --model_dir runs/gqp_demo/hemo \
  --test_json datasets/jsonl/hemo/val.jsonl \
  --out_dir outputs/attention/hemo

python scripts/diagnostics/counterfactual/eval_ism_cse.py \
  --model_dir runs/gqp_demo/hemo \
  --jsonl datasets/jsonl/hemo/val.jsonl \
  --out_dir outputs/cse/hemo
```

The resulting files include attention-mass summaries, controlled substitution effect heatmaps, and residue-level intervenability bar plots used to support the interpretation analyses.

References

[1] Chakradhar Guntuboina, Adrita Das, Parisa Mollaei, Seongwon Kim, and Amir Barati Farimani. Peptidebert: A language model based on transformers for peptide property prediction. The Journal of Physical Chemistry Letters, 14(46):10427–10434, 2023.

[2] Srivathsan Badrinarayanan, Chakradhar Guntuboina, Parisa Mollaei, and Amir Barati Farimani. Multi-peptide: multimodality leveraged language-graph learning of peptide properties. Journal of Chemical Information and Modeling, 65(1):83–91, 2024.

[3] Siyuan Wang, Michael T Bergman, Carol K Hall, and Fengqi You. De novo design of multiple microplastic-binding peptides with a protein language model-guided generative adversarial network. Journal of Chemical Information and Modeling, 65(16):8527–8537, 2025.

[4] Jacob Devlin, Ming-Wei Chang, Kenton Lee, and Kristina Toutanova. BERT: pre-training of deep bidirectional transformers for language understanding. CoRR, abs/1810.04805, 2018. URL http://arxiv.org/abs/1810.04805.

[5] Yinhan Liu, Myle Ott, Naman Goyal, Jingfei Du, Mandar Joshi, Danqi Chen, Omer Levy, Mike Lewis, Luke Zettlemoyer, and Veselin Stoyanov. Roberta: A robustly optimized BERT pretraining approach. CoRR, abs/1907.11692, 2019. URL http://arxiv.org/abs/1907.11692.

[6] Zeming Lin, Halil Akin, Roshan Rao, Brian Hie, Zhongkai Zhu, Wenting Lu, Nikita Smetanin, Robert Verkuil, Ori Kabeli, Yaniv Shmueli, et al. Evolutionary-scale prediction of atomic-level protein structure with a language model. Science, 379(6637):1123–1130, 2023.

[7] Ahmed Elnaggar, Michael Heinzinger, Christian Dallago, Ghalia Rehawi, Yu Wang, Llion Jones, Tom Gibbs, Tamas Feher, Christoph Angerer, Martin Steinegger, DEBSINDHU BHOWMIK, and Burkhard Rost. Prottrans: Towards cracking the language of life's code through self-supervised deep learning and high performance computing. bioRxiv, 2020. doi: 10.1101/2020.07.12.199554. URL https://www.biorxiv.org/content/early/2020/07/21/2020.07.12.199554.

[8] Sarthak Jain and Byron C Wallace. Attention is not explanation. In Proceedings of the 2019 Conference of the North American Chapter of the Association for Computational Linguistics: Human Language Technologies, Volume 1 (Long and Short Papers), pages 3543–3556, 2019.

[9] Sofia Serrano and Noah A Smith. Is attention interpretable? In Proceedings of the 57th Annual Meeting of the Association for Computational Linguistics, pages 2931–2951, 2019.

[10] Jules DP Valentin, Hervé Straub, Franziska Pietsch, Marion Lemare, Christian H Ahrens, Frank Schreiber, Jeremy S Webb, Henny C Van der Mei, and Qun Ren. Role of the flagellar hook in the structural development and antibiotic tolerance of pseudomonas aeruginosa biofilms. The ISME Journal, 16(4): 1176–1186, 2022.

[11] Surag Nair, Avanti Shrikumar, Jacob Schreiber, and Anshul Kundaje. fastism: performant in silico saturation mutagenesis for convolutional neural networks. Bioinformatics, 38(9):2397–2403, 2022.

[12] Tanja Kortemme, David E Kim, and David Baker. Computational alanine scanning of protein-protein interfaces. Science's STKE, 2004(219):pl2–pl2, 2004.

[13] Melissa D Boersma, Jack D Sadowsky, York A Tomita, and Samuel H Gellman. Hydrophile scanning as a complement to alanine scanning for exploring and manipulating protein–protein recognition: application to the bim bh3 domain. Protein Science, 17(7):1232–1240, 2008.
